# Copper(II) Complexes with Mixed Heterocycle Ligands as Promising Antibacterial and Antitumor Species

**DOI:** 10.3390/molecules25173777

**Published:** 2020-08-19

**Authors:** Arpad Mihai Rostas, Mihaela Badea, Lavinia L. Ruta, Ileana C. Farcasanu, Catalin Maxim, Mariana Carmen Chifiriuc, Marcela Popa, Mirela Luca, Natasa Celan Korosin, Romana Cerc Korosec, Mihaela Bacalum, Mina Raileanu, Rodica Olar

**Affiliations:** 1 National Institute of Materials Physics, 405A Atomiştilor Str., 077125 Măgurele-Ilfov, Romania; arpad.rostas@infim.ro; 2 Department of Inorganic Chemistry, Faculty of Chemistry, University of Bucharest, 90–92 Panduri Str., 050663 Bucharest, Romania; mihaela.badea@chimie.unibuc.ro (M.B.); catalin.maxim@chimie.unibuc.ro (C.M.); mirela_luk94@yahoo.com (M.L.); 3 Department of Organic Chemistry, Biochemistry and Catalysis, Faculty of Chemistry, University of Bucharest, 90–92 Panduri Str., 050663 Bucharest, Romania; lavinia.ruta@gmail.com (L.L.R.); ileana.farcasanu@chimie.unibuc.ro (I.C.F.); 4 Department of Microbiology, Faculty of Biology, University of Bucharest, 1–3 Aleea Portocalelor Str., 060101 Bucharest, Romania; carmen_balotescu@yahoo.com (M.C.C.); bmarcelica@yahoo.com (M.P.); 5 Faculty of Chemistry and Chemical Technology, University of Ljubljana, Večna pot 113, 1000 Ljubljana, Slovenia; natasa.celan@fkkt.uni-lj.si (N.C.K.); romana.cerc-korosec@fkkt.uni-lj.si (R.C.K.); 6 Department of Life and Environmental Physics, Horia Hulubei National Institute for Physics and Nuclear Engineering, 30 Reactorului Str., 077125 Măgurele-Ilfov, Romania; bmihaela@nipne.ro (M.B.); mina.raileanu@nipne.ro (M.R.); 7 Department of Electricity, Solid State and Biophysics, Faculty of Physics, University of Bucharest, 405A Atomiştilor Str., 077125 Măgurele-Ilfov, Romania

**Keywords:** Copper(II) complex, 1,2,4-triazolo[1,5-*a*]pyrimidine, Cytotoxicity, Biofilm, Metallonuclease activity, DNA intercalation

## Abstract

Complexes with mixed ligands [Cu(N-N)_2_(pmtp)](ClO_4_)_2_ ((**1**) N-N: 2,2′-bipyridine; (**2**) L: 1,10-phenanthroline and pmpt: 5-phenyl-7-methyl-1,2,4-triazolo[1,5-*a*]pyrimidine) were synthesized and structurally and biologically characterized. Compound (**1**) crystallizes into space group *Pa* and (**2**) in *P*-1. Both complexes display an intermediate stereochemistry between the two five-coordinated ones. The biological tests indicated that the two compounds exhibited superoxide scavenging capacity, intercalative DNA properties, and metallonuclease activity. Tests on various cell systems indicated that the two complexes neither interfere with the proliferation of *Saccharomyces cerevisiae* or BJ healthy skin cells, nor cause hemolysis in the active concentration range. Nevertheless, the compounds showed antibacterial potential, with complex (**2**) being significantly more active than complex (**1**) against all tested bacterial strains, both in planktonic and biofilm growth state. Both complexes exhibited a very good activity against B16 melanoma cells, with a higher specificity being displayed by compound (**1**). Taken together, the results indicate that complexes (**1**) and (**2**) have specific biological relevance, with potential for the development of antitumor or antimicrobial drugs.

## 1. Introduction

The copper species are unique both from the point of view of coordinative chemistry and from their biological relevance. Some features, such as stereochemical and oxidation state versatility or acid borderline character, strongly recommend the use of copper as central ion in complexes with a large variety of ligands, structures and properties.

Copper complexes have been the focus of intense studies aiming to design and develop valuable species with antitumor, anti-inflammatory or antimicrobial activities. Most of these complexes contain mixed ligands, e.g., N-N-chelating heterocycle such 2,2′-bipyridine (bpy) and 1,10-phenanthroline (phen), both chosen for their chelating ability and intercalative properties.

Several such complexes that exhibit potential antitumor and/or DNA cleavage abilities have been reported. For example, complexes with ancillary ligands (an N-N-chelating heterocycle and a bioactive scaffold containing species [1,2,3,4,5,6,7]), antibiotics [8,9,10,11,12,13,14], or anti-inflammatory drugs [15,16,17,18] have been shown to have potent anticancer effect, in some cases even better than the anti-tumor activity of cisplatin [2,3,4].

Moreover, complexes with metallonuclease-like activity have been the subject of several studies. In particular, complexes with drugs as auxiliary ligands have been shown to strongly bind to calf-thymus DNA (CT DNA) in an intercalative mode, performing their oxidative cleavage through reactive oxygen species (ROS) [10,11,12,13,14,15,16,17,18,19,20].

In a separate line of research, it was found that copper(II) complexes with N-N-chelating heterocycle and sulfonamide antibiotics were active against *Mycobacterium tuberculosis* [21] and *Pseudomonas aeruginosa* [22] strains. In addition, complexes with phen and quinolone antibiotics [23,24] proved to be active on various *Escherichia coli* strains, showing promising potential for the development of effective antimicrobials against antibiotic-resistant strains.

However, despite these multiple biological effects, the number of Cu(II) complexes containing a triazolopyrimidine derivative in addition to N-N-chelating heterocycle ligands is surprisingly limited. Only three such complexes have been synthesized and structurally characterized so far, with 1,2,4-triazolo[1,5-*a*]pyrimidine (tp) and 5,7-dimethyl-1,2,4-triazolo[1,5-*a*]pyrimidine (dmtp) [25]. Among these, the species with tp were more active against *Trypanosoma cruzi* and *Leishmania peruviana* in comparison with antiparasitic reference drugs [26].

The biological and coordinative properties of triazolopyrimidine derivatives makes them good candidates for fine-tuning the reactivity and properties of [Cu(N-N)_2_]^2+^ motif in relation to biomolecules. In this context, we developed novel perchlorate copper(II) complexes containing an N-N-chelating heterocycle ligand (2,2′-bipyridine or 1,10-phenanthroline) and 5-phenyl-7-methyl-1,2,4-triazolo[1,5-*a*]pyrimidine (pmtp). To enhance both the thermodynamic and kinetic stability of the complexes and to modulate lipophilicity and non-covalent interactions with biomolecules, N-N-chelating heterocycle ligands were selected. For biological activity modulation through lipophilicity and non-covalent interactions, the triazolopyrimidine ligand was used. Single-crystal X-ray diffraction evidenced its ability to establish both hydrogen and π-π stacking interactions [27]. Moreover, for Cu(II) complexes with this ligand a good antimicrobial activity was evidenced for chloride [28], acetate [29] and perchlorate [30] species on a wide range of pathogenic strains, both in planktonic and biofilm growth state. Furthermore, the [Cu(pmtp)(CH_3_COO)_2_]·0.5H_2_O complex exhibits a dose-dependent antiproliferative effect on human colon adenocarcinoma (HT 29) cell and an anti-inflammatory effect similar with diclofenac [29].

Single-crystal X-ray analyses, as well as different spectroscopic methods, were used in order to characterize the complexes. Data concerning evaluation of complexes for their interaction with some eukaryotic cells are also reported.

## 2. Results and Discussion

**Scheme 1:**
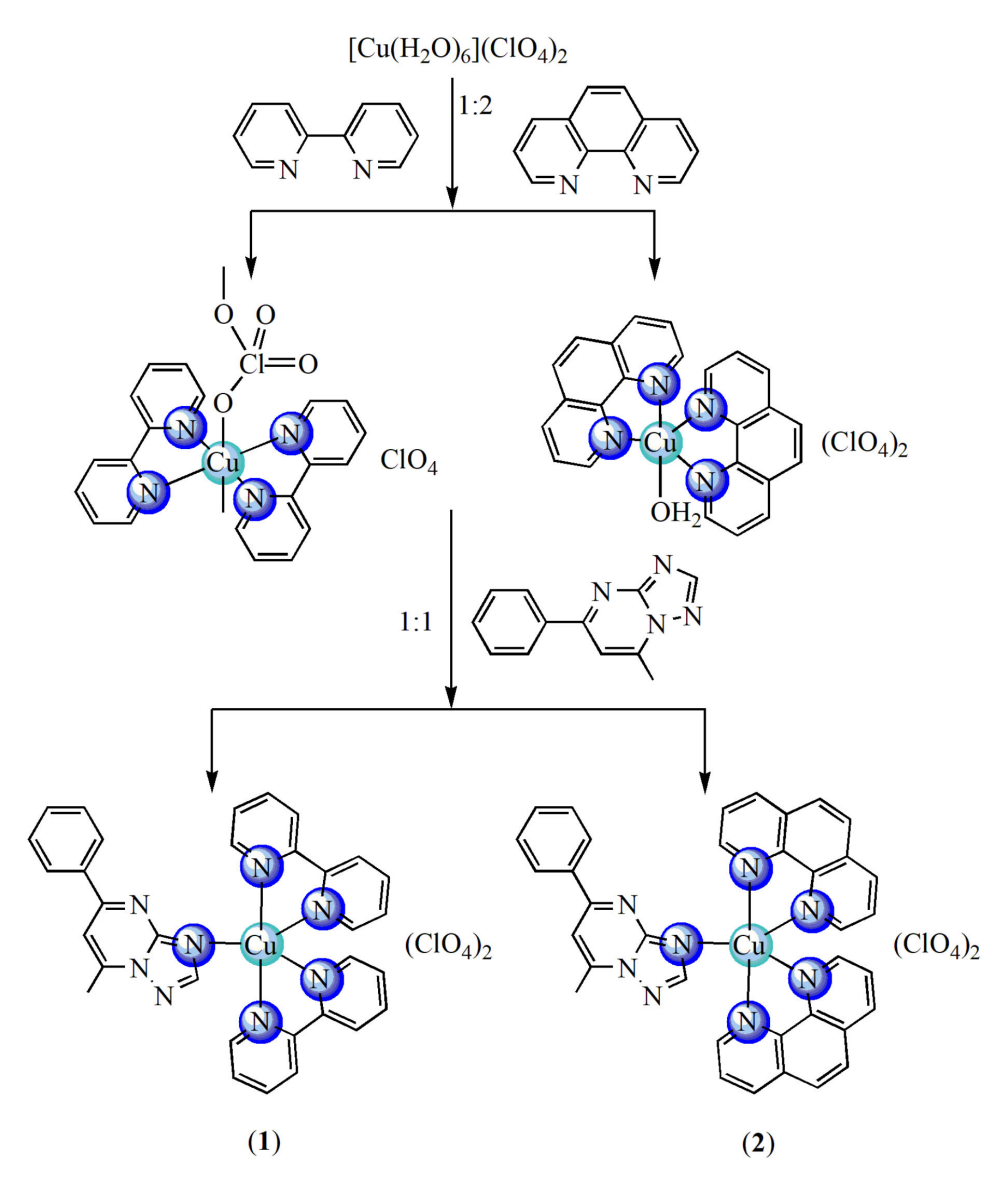
The route of complexes synthesis.

A new series of copper(II) complexes with mixed ligands 2,2′-bipyridine (bpy) or 1,10-phenanthroline (phen) and 5-phenyl-7-methyl-1,2,4-triazolo[1,5-*a*]pyrimidine (pmtp) was synthesized by stepwise reactions as depicted in Scheme 1. First, the [Cu(N-N)_2_]^2+^ intermediates were prepared and then the pmtp was added in suitable proportions. The compounds were characterized as mononuclear species by single-crystal X-ray diffraction, elemental and thermal analysis, EPR, FTIR and UV-Vis spectra.

### 2.1. Description of the Crystal Structure of Cu(II) Complexes

The structures of both complexes were solved by single-crystal X ray diffraction, and a summary of crystallographic data is presented in Table 1.

**Figure 1:**
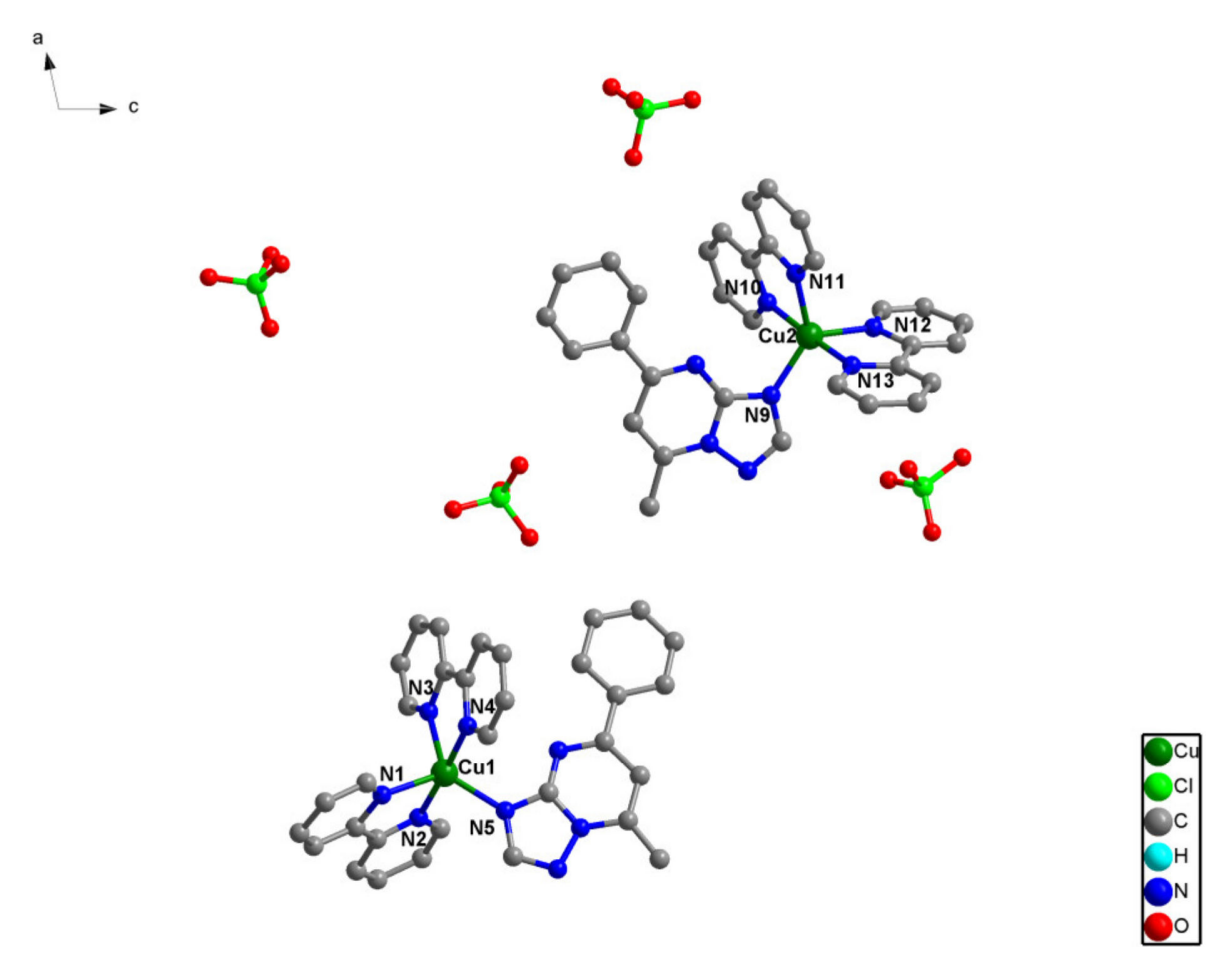
The asymmetric unit of two crystallographically non-equivalent mononuclear [Cu(bpy)_2_(pmtp)](ClO_4_)_2_ (**1**) complex along b axes.

The crystal structure of (**1**), [Cu(bpy)_2_(pmtp)](ClO_4_)_2_, consists of cationic mononuclear species and perchlorate anions (Figure 1). The asymmetric unit contains two crystallographically non-equivalent mononuclear units that show similar coordination geometries. The copper ion is pentacoordinated with a trigonal bipyramidal stereochemistry, with a continuous shape measure (CShM) value of 0.891 for Cu1 and 0.957 for Cu2 (Supplementary Table S1). The trigonal plane is formed by two nitrogen atoms from two bpy ligands (Cu1–N1 = 1.971(15); Cu1–N3 = 2.072(18); Cu2–N11 = 2.056(16); Cu2–N12 = 2.071(17) Å), and another nitrogen atom from pmtp ligand (Cu1–N5 = 2.013(14); Cu2–N9 = 1.995(15) Å). The axial positions are occupied by two nitrogen atoms from two different bpy ligands (Cu1–N2 = 1.971(15); Cu1–N4 = 1.968(16) Å; Cu2–N10 = 1.987(15); Cu2–N13 = 1.958(15) Å). The intramolecular Cu1···Cu2 distance is 15.999 Å.

Selected bond distances and angles for the two compounds are collected in Table 2. The phenyl and 1,2,4-triazolo[1,5-*a*]pyrimidine rings within the pmtp ligand are not coplanar (dihedral angles: 9.937° for one pmtp molecule and 11.40° for the other one).

**Figure 2:**
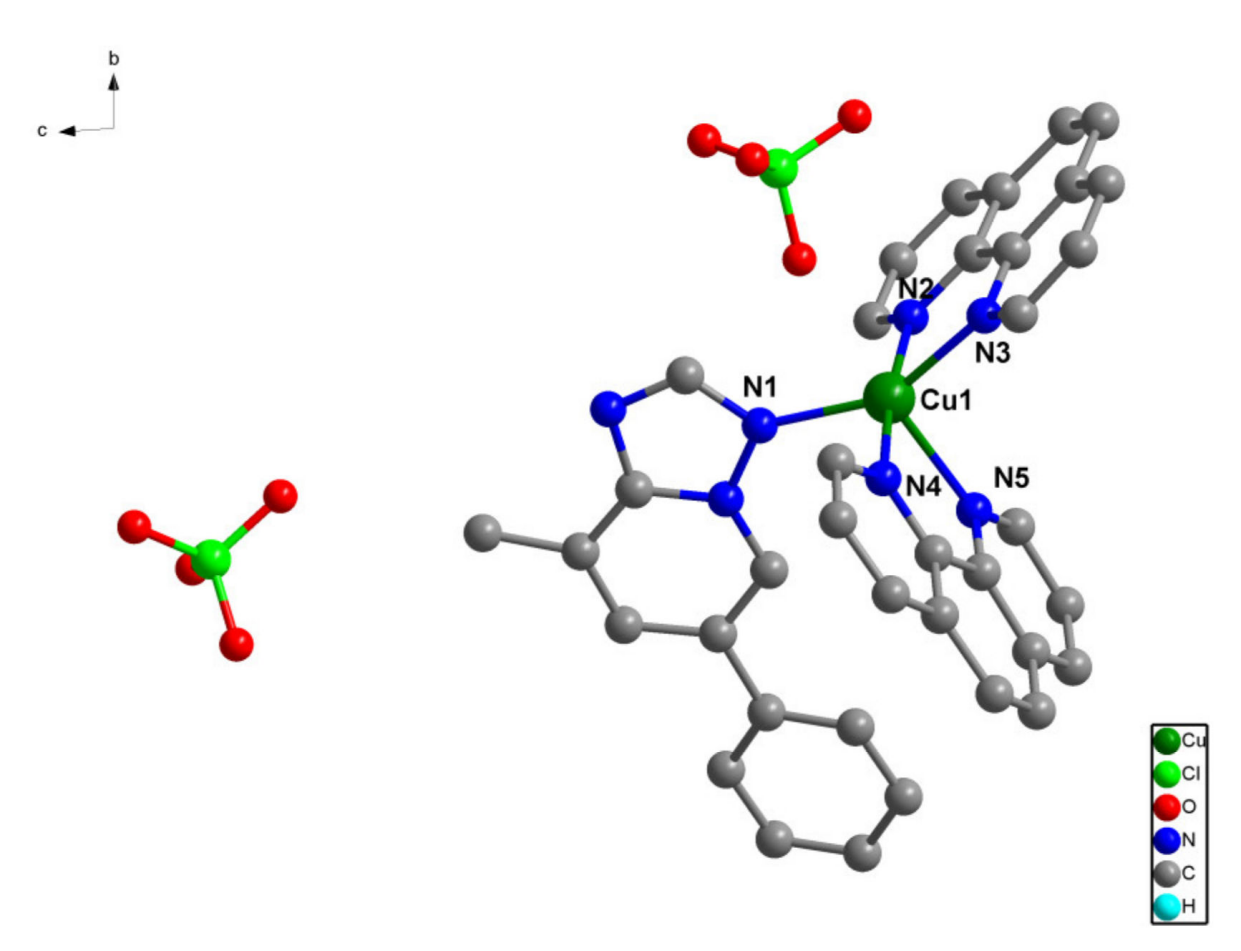
Crystal structure of [Cu(phen)_2_(pmtp)](ClO_4_)_2_ (**2**) along a axes.

The structure of complex (**2**) is made up of cationic mononuclear entities [Cu(phen)_2_(pmtp)]^2+^ and perchlorate anions (Figure 2). The crystallographically independent copper atoms exhibit a distorted trigonal bipyramidal geometry, with a CShM value of 2.277. Each metal atom is coordinated by four nitrogen atoms from two 1,10-phenanthroline ligands and one nitrogen atoms from a pmtp molecule. The Cu–N distances vary between 1.999(4) and 2.203(3), respectively (Table 2).

The analysis of the packing diagram reveals the formation of supramolecular dimers, through intermolecular π-π stacking interactions (carbon-carbon distances varying between 3.46 and 3.83 Å) established between pmtp ligands (Supplementary Figure S1). Further, these supramolecular dimers interact through intermolecular π-π stacking interactions established between 1,10-phenanthroline ligands, resulting in supramolecular chains (Supplementary Figure S2).

### 2.2. Physico-Chemical Characterization of Complexes

#### 2.2.1. FT-IR Spectra

In comparison with both ligands’ spectra, in the corresponding complexes three bands assigned to ν(C=N) mode were observed in the 1580–1640 cm^−1^ range. In the complexes’ spectra, the band associated with the triazolopyrimidine core (1603 and 1605 cm^−1^) is shifted to lower wavenumbers in comparison with the free ligand (1612 cm^−1^). This behavior was also observed for other complexes with pmtp coordinated through N^3^ from triazole ring [28,29,30]. The characteristic bands for overlapping ν(C=N) and ν(C=C) stretching vibrations of N-N-chelating heterocycle ring appear in the 1625–1635 cm^−1^ range. Perchlorate function of a free ion is confirmed by the presence of the characteristic bands about 1190 and 625 cm^−1^ arising from ν_3_(ClO_4_) and ν_4_(ClO_4_) stretching vibrations, respectively [31]. The low intensity bands at 417 and 442 cm^−1^ were assigned to the ν(Cu-N) stretching vibration.

#### 2.2.2. UV-Vis Spectra

The diffuse reflectance spectra, in the UV-Vis range, of the Cu(II) complexes show absorption bands that are characteristic to penta-coordinated copper(II) ion in a trigonal bipyramidal major stereochemistry [32]. Thus, the absorption maximum at 615/610 nm was assigned to d_xz_, d_yz_→d_z2_ transition, while the shoulder at higher wavelength is due to d_xy_→d_z2_ transition for a CuN_5_ chromophore with an intermediate stereochemistry between trigonal bipyramidal and square-pyramidal one. As a result of coordination, the band assigned to intraligand π→π* transitions for both heterocycle ligands is overlapped and appears at 290/265 nm while the band assigned to n→π* transition for N-N-chelating ligand appears around 350 nm (Supplementary Figure S3).

#### 2.2.3. EPR Spectroscopy

##### Solid-State EPR Spectroscopy

**Figure 3:**
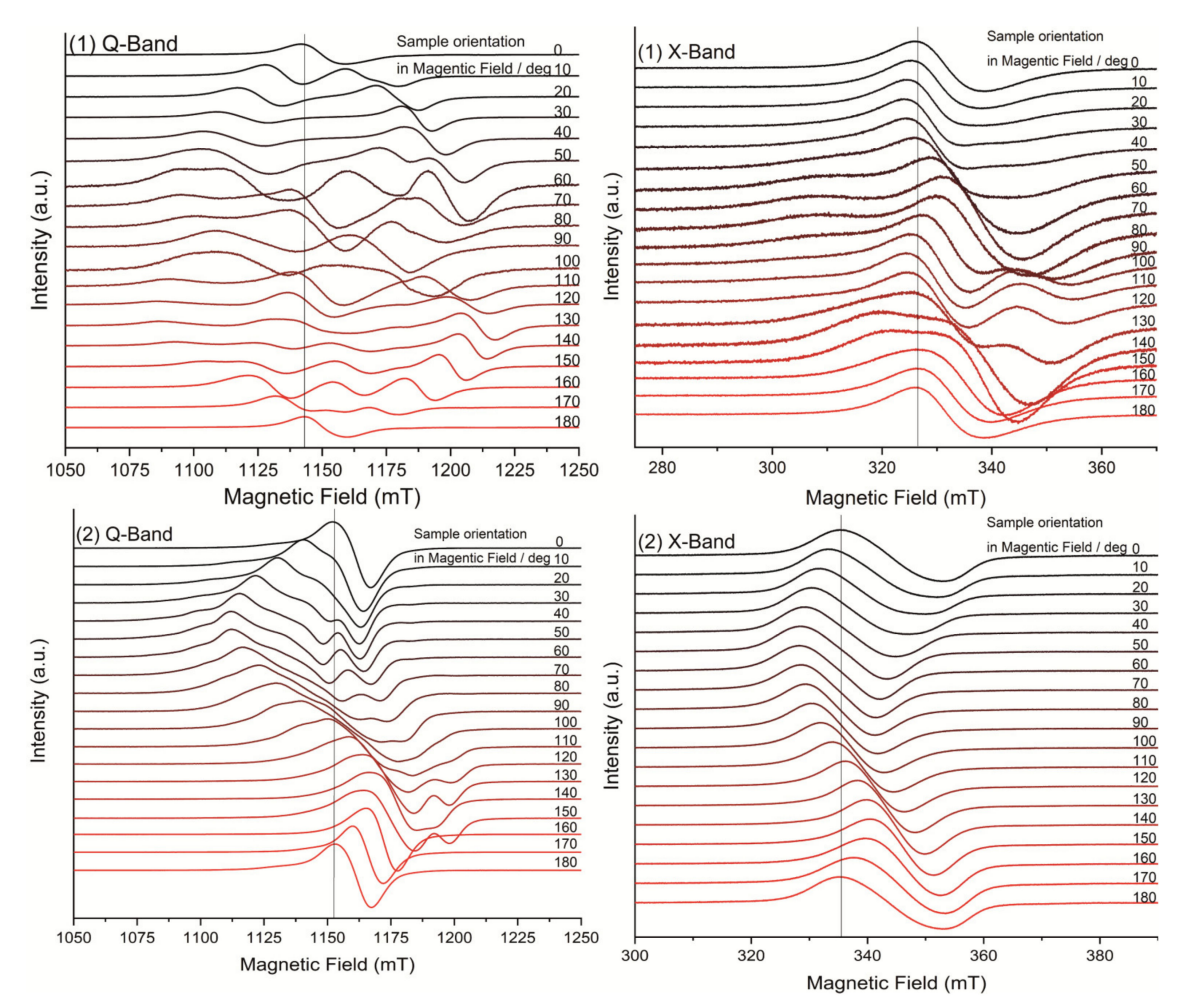
EPR spectra in Q-band and X-band with rotation along the main magnetic field of complex (**1**) (**top**) and complex (**2**) (**bottom**).

The EPR spectra obtained in the ab crystal plane at X- and Q-band and room temperature are shown in Figure 3. A single Lorentzian-shaped resonance line, characteristic of strong exchange, was observed for a magnetic field orientation at 0 and 180° at both microwave frequencies, which is in good agreement with the results regarding the structure of the complexes. In contrast, spectra with partially solved hyperfine structure, typical of weak exchange, were obtained for field orientations in the range of 20 to 160°. As one can see the EPR spectra are highly complex. The spectral feature is clearly rhombic, but the g tensor values cannot be estimated because of the complex hyperfine structure that overlaps with the strong spin–spin interaction.

##### Solution EPR Spectroscopy

The intensity and the line broadening of the DMSO solution of complexes EPR spectra were monitored over one week in order to obtain information concerning their stability. The results indicate that after one week, the EPR spectra of both compounds did not change significantly, revealing a very high stability under atmospheric conditions of the tested compounds (Supplementary Figure S4). Moreover, the g tensor values in solution of 2.232, 2.053 and 2.088 for (**1**) and 2.23, 2.051 and 2.076 for (**2**) resulting from the simulated spectra (Supplementary Figure S5) give isotropic g-values g_iso1_ = 2.124 and g_iso2_ = 2.123. These values are similar to the g-values of the samples oriented parallel (0°) to the main magnetic field in solid g_1_ = 2.122 and g_2_ = 2.12, where due to the strong exchange interaction, only the isotropic g-value is observable. These aspects reveal the fact that the complex coordination was preserved in solution. This high kinetic stability can be attributed to both the stable five-membered chelate rings formed with the N-N-chelating heterocycle and the bulky ligand pmtp, which protects the core of the complexes from substitution and/or solvent coordination processes.

#### 2.2.4. Thermal Analysis

The thermal behavior of the complexes was investigated in air by TG-DSC technique coupled with MS analysis of the evolved gases. Considering the rapid decomposition of the complexes, very small quantities of the sample were used.

Both complexes are quite stable, since decomposition starts at 198 and 195 °C, respectively (Supplementary Figure S6, Table S2). The simultaneous TG-DSC curves for complex (**1**) indicate a three-step decomposition pattern in which heat is released at each step.

All steps consist of a superposition of multiple processes according to products identified in the gaseous mixture (Supplementary Figure S7). The decomposition starts with the fragmentation of both organic ligands overlapped with oxidative degradation of some moieties and a part of perchlorate decomposition. These transformations give rise to CH_3_ (*m/z* 15), C_6_H_5_ (*m/z* 77), C_5_H_6_N (*m/z* 80), C_2_H_5_N_4_ (*m/z* 85) and C_5_H_8_N_3_ (*m/z* 110) as species resulting from both organic species fragmentation as well as H_2_O (*m/z* 18), CO_2_ (*m/z* 44), ClO (*m/z* 51), Cl (*m/z* 35) and other chlorine-based derivatives as products of oxidative degradation and perchlorate decomposition. The perchlorate decomposition together with oxidative degradation proceeds also in the next steps. As a result, nitrogen-containing fragment NO (*m/z* 30) was identified, along with those corresponding to chlorine-containing fragments, in the gaseous mixture to be eliminated at up to 660 °C. The overall mass loss corresponds to CuO as the final product (found/calcd.: 90.83/89.87%).

The thermal transformations of complex (**2**) occur in four steps accompanied by exothermic effects. Fragments such as Cl (*m/z* 35), ClO (*m/z* 51) and Cl_2_ (*m/z* 70), identified in the gaseous products at all steps, indicate that a part of perchlorate is decomposed in each one up until the final one. This process is accompanied by organic part fragmentation as the main process in the first steps and by oxidative degradation in the last ones. The last steps are accompanied by NO (*m/z* 30) production as result of the oxidative degradation of nitrogen-containing moieties. The found/calculated overall mass loss of 90.74/90.45% is in accordance with the compound formula.

### 2.3. Biological Characterization

#### 2.3.1. Superoxide Scavenging Ability

The compounds’ ability to scavenge reactive oxygen species was tested by EPR spectroscopy. Supplementary Figure S8 shows the EPR signal evolution as a function of the concentration of added O_2_^−^ to a 2.5 μM complex (**1**) and (**2**) DMSO solution, respectively. The nominal concentration of O_2_^-^ was 10, 20 and 30 µM, respectively. Because the O_2_^−^ is highly reactive, this concentration can vary slightly. The EPR spectra show a clear decrease in the signal, indicating that both complexes trap the superoxide radicals (Figure S8). Moreover, the Cu(II) to Cu(I) reduction was indicated by the color changing from blue to yellow, a color that would remain stable for more than one week.

#### 2.3.2. Effect of Compounds on Saccharomyces Cerevisiae Cells

The toxicity of compounds (**1**) and (**2**) was tested against *Saccharomyces cerevisiae* (“wild-type”, WT) laboratory strain BY4741. It was observed that within the range 0.1–0.2 mM (final concentration in liquid YPD), the two compounds did not significantly affect cell proliferation when compared to cells grown in the absence of compounds (Figure S9A). Nevertheless, both compounds became toxic at higher concentrations, with IC_50_ (0.48 ± 0.16) mM for (1) and (0.58 ± 0.21) mM for (**2**), respectively. Compounds (**1**) and (**2**) were even more toxic when yeast cells lacked the Cu/Zn-superoxide dismutase Sod1 (sod1Δ cells) or Mn-superoxide dismutase Sod2 (sod2Δ cells) (Figure S9B). The increased sensitivity of sod1Δ and sod2Δ cells could not be correlated with an increased complex uptake, as the WT, sod1Δ and sod2Δ strains were not significantly different in terms of compounds uptake, as seen indirectly by determining the total copper cellular content (Figure S9C).

#### 2.3.3. DNA Binding Properties of Compounds

As copper complexes often exert their biological activity through interaction with DNA, we investigated whether the toxicity of (**1**) and (**2**) could be related to their capacity to bind to DNA. To test this possibility, the modifications induced by DNA to spectral characteristics of the two complexes were determined through both absorption and fluorescence spectra modifications.

##### Absorption Spectra Modifications

**Figure 4:**
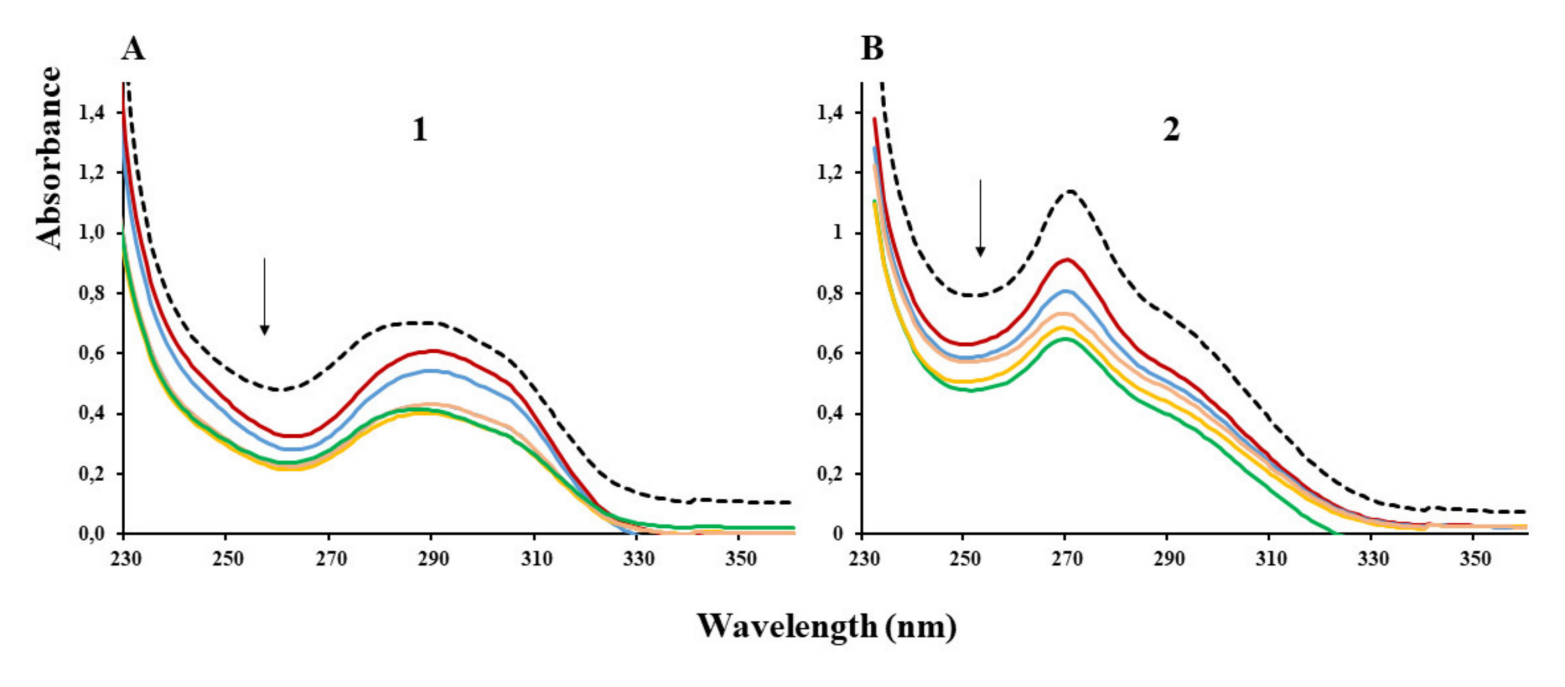
Absorption spectra of (**1**) (**A**) and (**2**) (**B**) in the absence of DNA (black dotted line) or in the presence of increasing DNA concentration (colored lines) (arrow indicates the directionality of [DNA] increase for 3 µM complex concentration).

Absorption spectroscopy represents an invaluable tool for studying any compound interaction with DNA through band intensity modification, which generally indicates intercalative abilities [33]. The binding ability of complexes (**1**) and (**2**) to λ-DNA in buffer solution was determined by their effect on the UV absorption spectra by using a fixed complex concentration (3 μM), to which an increasing volume of DNA solution was added. The binding of complex (**1**) to λ-DNA led to a decrease in the absorption intensity of the band located at 290 nm (Figure 4A), indicating an intercalation of (**1**) into λ-DNA strands. Reaching saturation was accompanied by a slight hypsochromic shift (Figure 4A, green spectrum), which became more evident with increasing λ-DNA concentration (data not shown, for the sake of clarity). The binding of complex (**2**) to λ-DNA was also accompanied by a decrease in the absorption intensity of the spectral bands with DNA concentration increasing (Figure 4B). The hypochromism observed for the band at 270 nm indicates that similarly to (**1**), complex (**2**) also binds to λ-DNA, probably by intercalation through the planar aromatic rings of the N-N-chelating ligands [34]. Increasing λ-DNA after reaching saturation (Figure 4B, green spectrum) was no longer accompanied by any effect, and the spectra were not significantly different from that shown as a green line (data not shown).

##### Fluorescence Spectra

**Figure 5:**
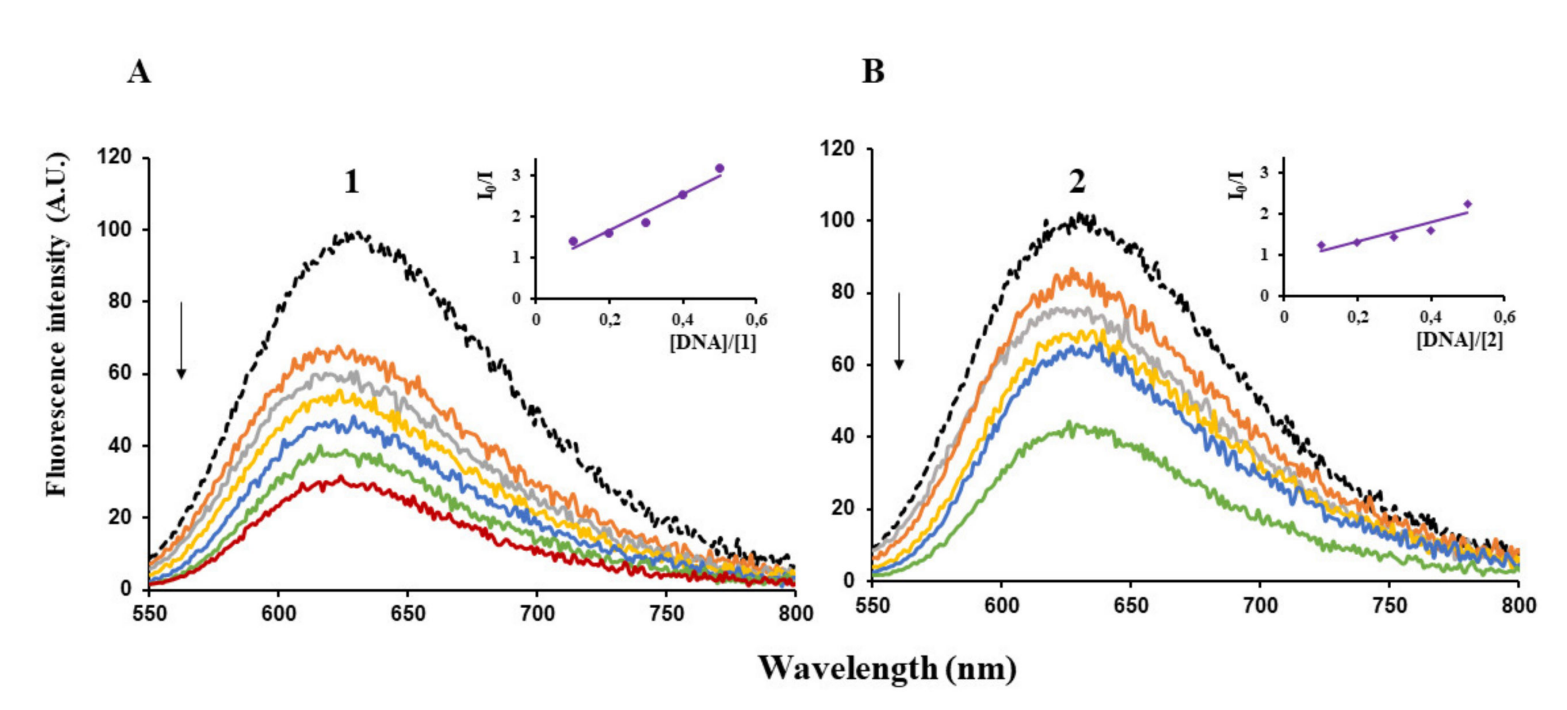
Emission spectra of λ-DNA/EB in Tris/HCl/NaCl buffer, pH = 8, in the absence (black curves) or in the presence of increasing concentrations of (**1**) (**A**) and (**2**) (**B**) (arrows indicate increase in [complex]. Insets: plots of emission intensity versus [DNA]/[Complex]. I_0_ = fluorescence intensity in the absence of complex. I = fluorescence intensity in the presence of complex).

Furthermore, the capacity of (**1**) and (**2**) to quench the fluorescence of λ-DNA/EB adduct was determined. Ethidium bromide (EB) is one of the most used intercalating dye for DNA detection as it causes intense fluorescence after intercalating into DNA [35,36]. When a complex intercalates into DNA, it is expected that the fluorescence of the λ-DNA/EB adduct decreased. We noticed indeed that when adding increments of (**1**) or (**2**) to λ-DNA pre-treated with EB, the fluorescence intensity decreased, a clear indication that the complex competes with EB for the binding sites of DNA (Figure 5).

#### 2.3.4. Nuclease Activity

**Figure 6:**
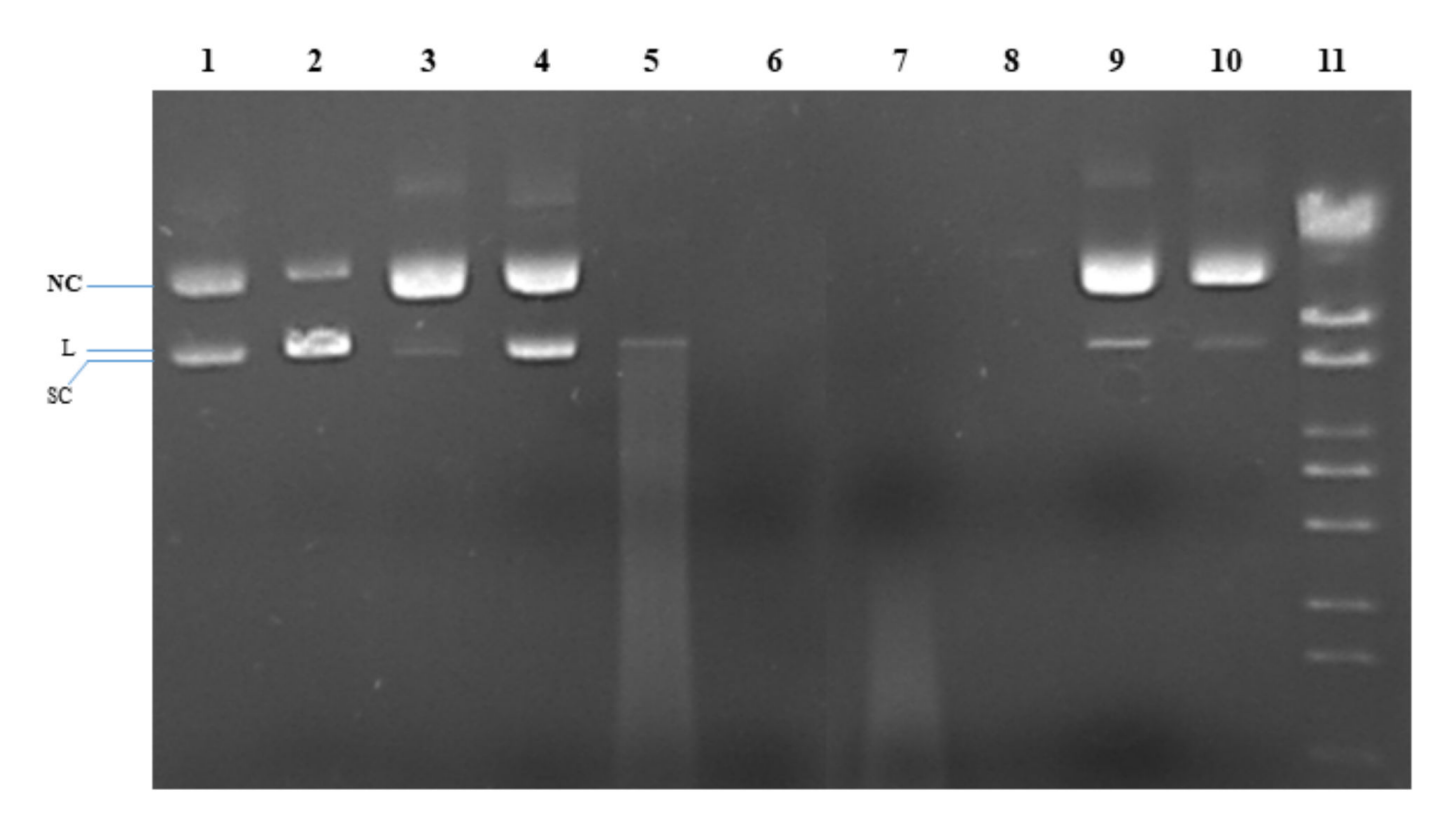
Gel electrophoresis image of pRSII425 (100 ng/μL) after incubation for 1 h at 37 °C with the compounds, except for *Eco*RI (5 min). Line 1: pRSII325; Line 2: pRSII325/*Eco*RI (partial digestion); Line 3: pRSII325 + 1 mM ascorbate; Line 4: pRSII325 + 1 mM H_2_O_2_; Line 5: pRSII325 + 1 mM ascorbate + 3 µM (**1**); Line 6: pRSII325 + 1 mM ascorbate + 3 µM (**2**); Line 7: pRSII325 + 1 mM H_2_O_2_ + 3 µM (**1**); Line 8: pRSII325 + 1 mM H_2_O_2_ + 3 µM (**2**); Line 9: pRSII325 + 3 µM (**1**); Line 10: pRSII325 + 3 µM (**2**); Line 11: molecular marker (λDNA/*Sty*I).

The nuclease activity of copper(II) complexes (**1**) and (**2**) was tested on plasmid pRSII325, which is a 6835 bp circular plasmid. Gel electrophoresis of purified pRSII325 identifies two main bands belonging to the supercoiled (SC) plasmid, which migrates faster, and the nicked-circular (NC) plasmid, which migrates slower (Figure 6, first line). The linear plasmid (L) migrates slightly slower than the SC form (Figure 6, line 2, showing pRSII325 partially cleaved with *Eco*RI). It was noted that both ascorbate (Figure 6, line 3) and H_2_O_2_ (Figure 6, line 4) caused DNA relaxation to the NC form. Compounds (**1**) and (**2**) also caused plasmid relaxation to the NC form (Figure 6, lines 9 and 10). The intensities of the DNA bands in the presence of (**1**) and (**2**) were different, tempting us to speculate that (**2**) may have an intrinsic nuclease activity. Incubating pRSII325 with (**1**) and (**2**) in the presence of either ascorbate (Figure 6, lines 5 and 6) or H_2_O_2_ (Figure 6, lines 7 and 8) led to DNA degradation, indicating a strong nuclease activity for both (**1**) and (**2**) in the presence of ascorbate or H_2_O_2_. In this case, the nuclease activity of (**2**) also appears stronger than the nuclease activity of (**1**), as suggested by the lack of degraded DNA smears in lines 6 and 8 (compared to lines 5 and 7).

It was observed that most of the complexes that showed an efficient DNA-cleavage activity display one or more of the following features: (i) ability to intercalate into DNA strands, (ii) a proper potential of Cu(II)/Cu(I) redox couple (in the range 0.3–0.9 V) suitable for both superoxide disproportionation and hydrogen peroxide reduction, (iii) presence of a fused aromatic ring system that stabilizes Cu(I) in the presence of activating agent, (iv) ligands that can be easily substituted or free coordinative sites, and (v) hydrophilic groups (coordinated or not) near to the metal center that can stabilize HO. As can be seen from the compound structure, complex (**2**), except for feature (ii), fulfills the other characteristics, so it is not surprising that it exhibits an intrinsic nuclease activity.

#### 2.3.5. Microbiological Assay

The antimicrobial activity of copper(II) complexes (**1**) and (**2**) was evaluated against several Gram-negative and Gram-positive bacterial strains, including the following resistant strains: methicillin-resistant *S. aureus* (MRSA), the extended spectrum beta-lactamase (ESBL) producing *E. coli* 5, and the multi-drug-resistant *P. aeruginosa* 9027 strain. The efficiency of the obtained complexes was assessed on bacterial strains in planktonic growth (MIC assay), as well as against biofilms formed by the respective strains (MBEC assay).

The MIC values (Table 3) indicate a better activity of the complexes in comparison with that of the pmtp, bpy and phen ligands for all tested strains, as revealed by the decreased MIC values of the complexes by 2 to 60 times. Complex (**2**) proved to be significantly more active than complex (**1**) against all tested strains. Both complexes were more active in comparison with intermediate {[Cu(bpy)_2_(μ_2_OClO_3_)]·ClO_4_}n (CuBP) and [Cu(phen)_2_(OH_2_)](ClO_4_)_2_ (CuPP) species, except complex (**1**) for which the activity was lower on all three *S. aureus* strains, and complex (**2**), with lower activity against the *E. coli* ESBL and one of the three *S. aureus* strains.

In the case of *E. coli* strains, the ligands exhibited the same efficiency against the susceptible and resistant strain, while the complexes proved to be more active against the *E. coli* 25922 susceptible strain compared to the ESBL producing *E. coli* strain. In the case of *P. aeruginosa,* the two complexes exhibited similar efficiency against the susceptible and resistant strain. For the three *S. aureus* strains, complex (**2**) was more efficient against both susceptible *S. aureus* 25923 and the MRSA ones. Although the activity on resistant strains was similar or comparable to that on the susceptible ones, it is worth mentioning the very low active concentration of complex (**2**) against the MRSA isolate.

It is well known that when bacteria are adhering to a surface and develop biofilms, they become much more resistant than when they grow in suspension as floating cells. In this context, it is remarkable that for the complexes (**1**) and (**2**), the minimum biofilm eradication concentration (MBEC) values were at least equal with the MIC values or even lower in case of *P. aeruginosa* 9027 and *S. aureus* 6538 biofilms (Table 4). In case of *E. coli* strains, the ligands, the intermediate complex and complex (**1**) exhibited the same efficiency against the susceptible and resistant strain, excepting the pmtp ligand, which was more active against the ESBL strains. In the case of complex (**2**), the ligand was more active against the biofilm formed by the ESBL strain, while the intermediate complex and complex (**2**) proved to be more active against the *E. coli* 25922 susceptible strain. In the case of *P. aeruginosa,* the ligands, as well as the two complexes, exhibited, remarkably, a better anti-biofilm activity against the resistant strain, while the intermediate complexes exhibited a similar efficiency against the susceptible and resistant strain. For the three *S. aureus* strains, complex (**2**) was by far more efficient against both susceptible and the MRSA strains. Although the activity on resistant strains was similar or comparable to that on susceptible ones, it is worth mentioning the very low active anti-biofilm concentrations of complex (**2**), as compared to intermediate complex and ligands. These results indicate the promising potential of these compounds as anti-biofilm agents. A good anti-biofilm ability was recently related to the nuclease activity observed for Cu(II) species formed in vitro with some host-defense peptides [37].

#### 2.3.6. Cytotoxicity Assays

The cytotoxicity of ligand and copper(II) complexes was tested on normal BJ cell as well as on B16 (melanoma) tumor cells, which were grown for 48 h in culture medium containing the tested complexes in 0–25 μg/mL concentration range.

**Figure 7:**
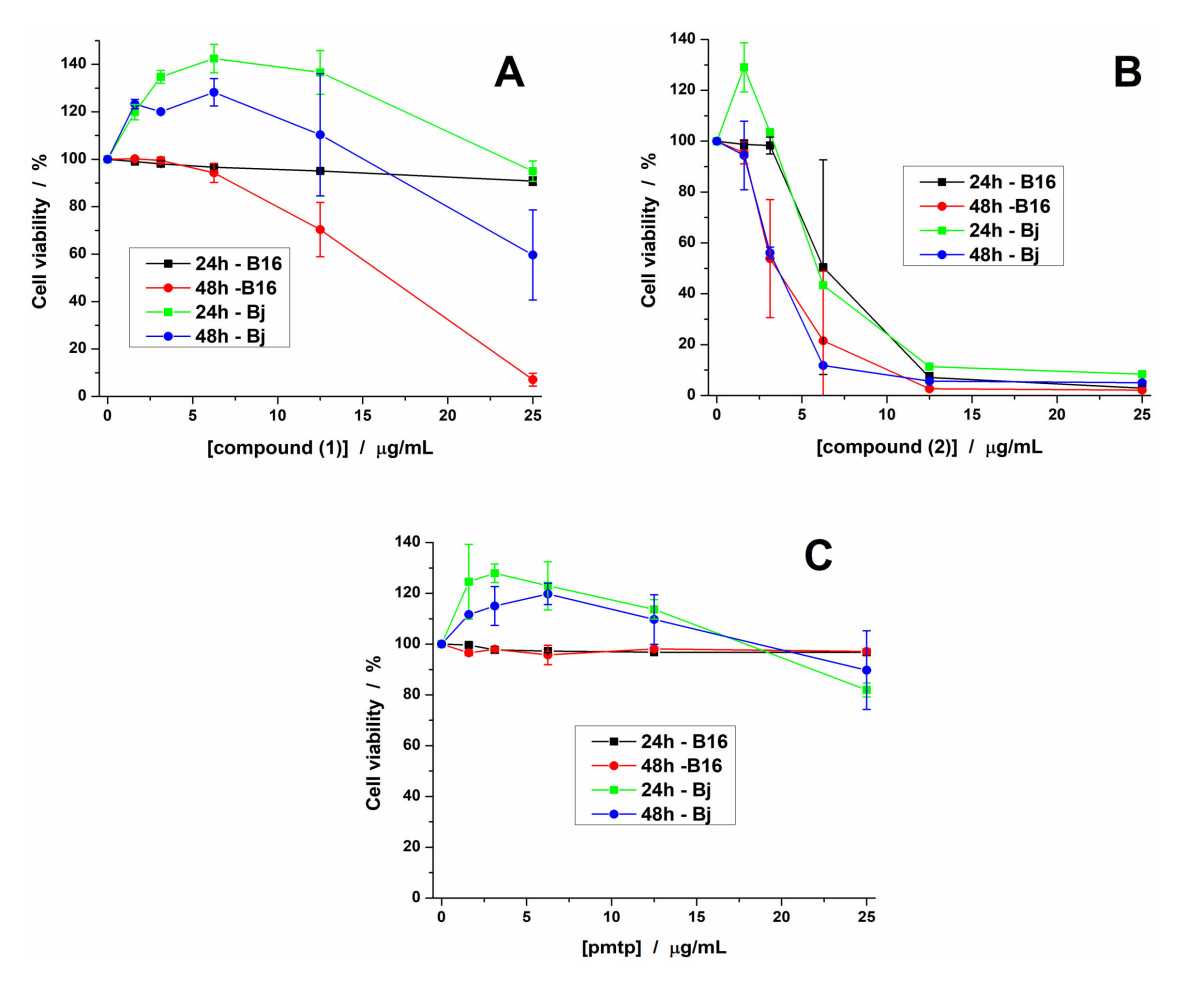
The cytotoxic effect of compounds (**1**) (**A**), (**2**) (**B**) and pmtp (**C**) on BJ and B16 cells evaluated at 24 and 48 h (each value represents the mean ± SD).

Complex (**1**) induced an increase in the viability of BJ cells at lower concentrations (up to 12 μg/mL) followed by a decrease in their viability up to 70% after 48 h of incubation in the presence of the highest tested concentration. Complex (**1**) did not affect the viability of B16 cells after 24 h of treatment, but induced a decrease of viability with increasing concentration after 48 h of treatment (Figure 7A), the IC_50_ at 48 h being 15.46 μg/mL (0.02 mM) (Table 5). These results show that complex (**1**) exhibits a different cytotoxic activity against normal versus tumor cells.

Complex (**2**) exhibits a stronger cytotoxic effect against both cell lines also after 24 h and 48 h of treatment (Figure 7B), even at the lowest tested concentration, with IC_50_ values ranging from 3.30 to 6.25 μg/mL (0.004 to 0.0075 mM). Unlike complexes, the pmtp did not affect the viability of the two cell lines at any of the tested concentrations (Figure 7C).

The hemolysis induced by the tested compounds at the highest concentrations used in the viability studies (25 µg/mL) was below the threshold of 5% established by ASTM F 756–00 [38], above which a substance is considered hemolytic. As observed from the results (Table 5), none of the tested compounds has hemolytic activity.

It is worth mentioning that for other copper compounds tested on melanoma cells the reported IC_50_ values were similar [39,40,41] or even higher [42] compared with the values found for complexes (**1**) and (**2**).

When considering the biomedical applications of different compounds, the therapeutic index (TI), a parameter indicating cell specificity of different drugs, is determined [43]. TI was calculated for each treatment time as the ratio of the IC_50_ value that produces a toxic effect on normal (BJ) and the IC_50_ value determined for tumor cells (B16). When no toxic effect was observed at the highest tested concentration, a value two times higher was used to calculate the TI. Taking into account the fact that a higher TI value indicates a higher specificity of the compound for the tumoral cells, the values presented in Table 5 reveal that complex (**1**) has a high affinity for tumor cells, with a TI value of 3.23, while for (**2**), no specificity was observed.

**Figure 8:**
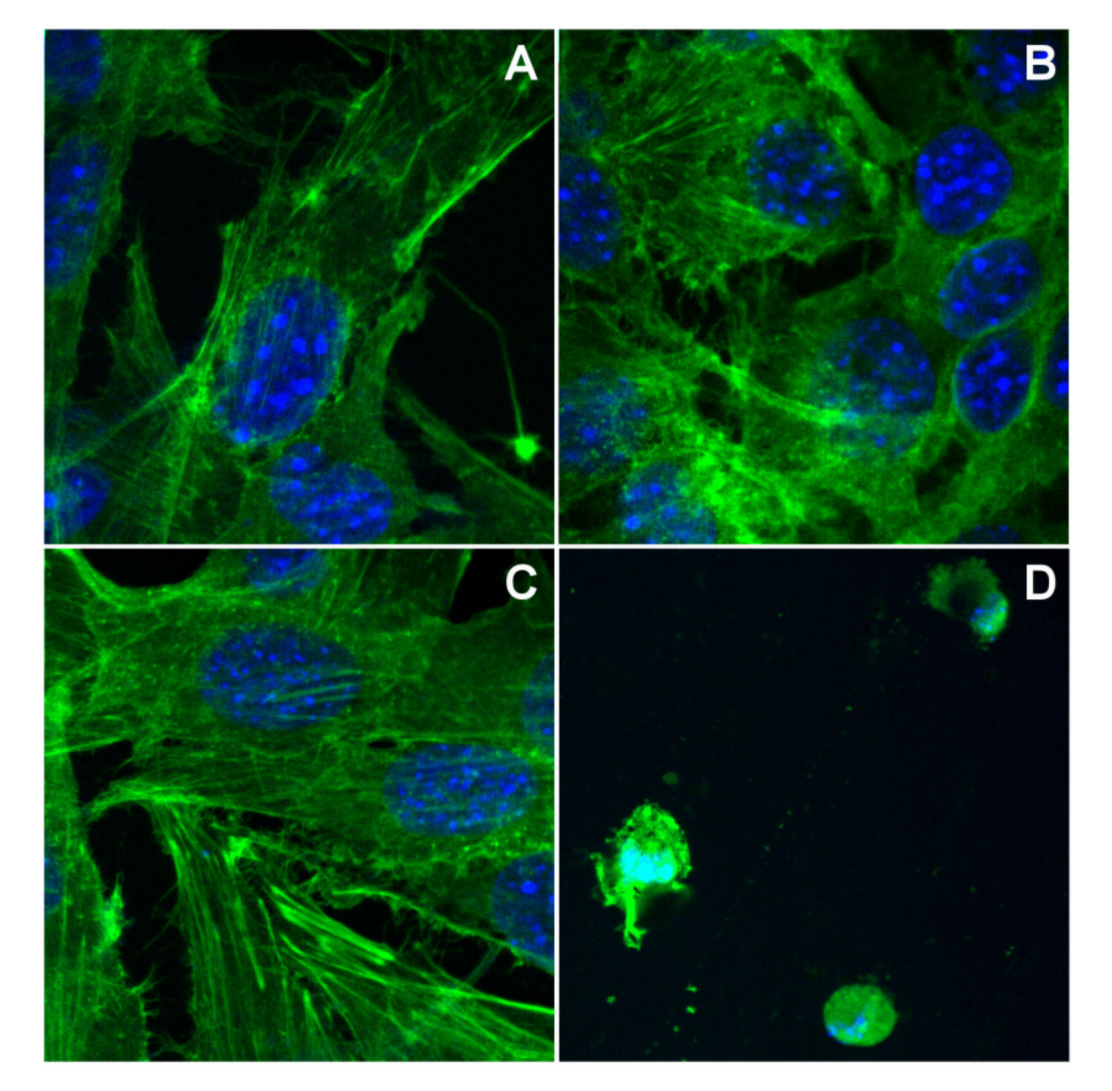
The cytoskeleton alteration for B16 cells: (**A**) control cells, (**B**) cells treated with pmtp, (**C**) cells treated with (**1**), (**D**) cells treated with (**2**).

The treatment of B16 cells for 24 h at a concentration of 6 μg/mL of pmtp and (**1**) does not alter the morphology of the cells as compared with the control ones, while complex (**2**) affects the cell morphology in the same conditions (Figure 8). Specifically, both cytoskeleton and nucleus are modified by altering the specific structure observed in normal cells.

**Figure 9:**
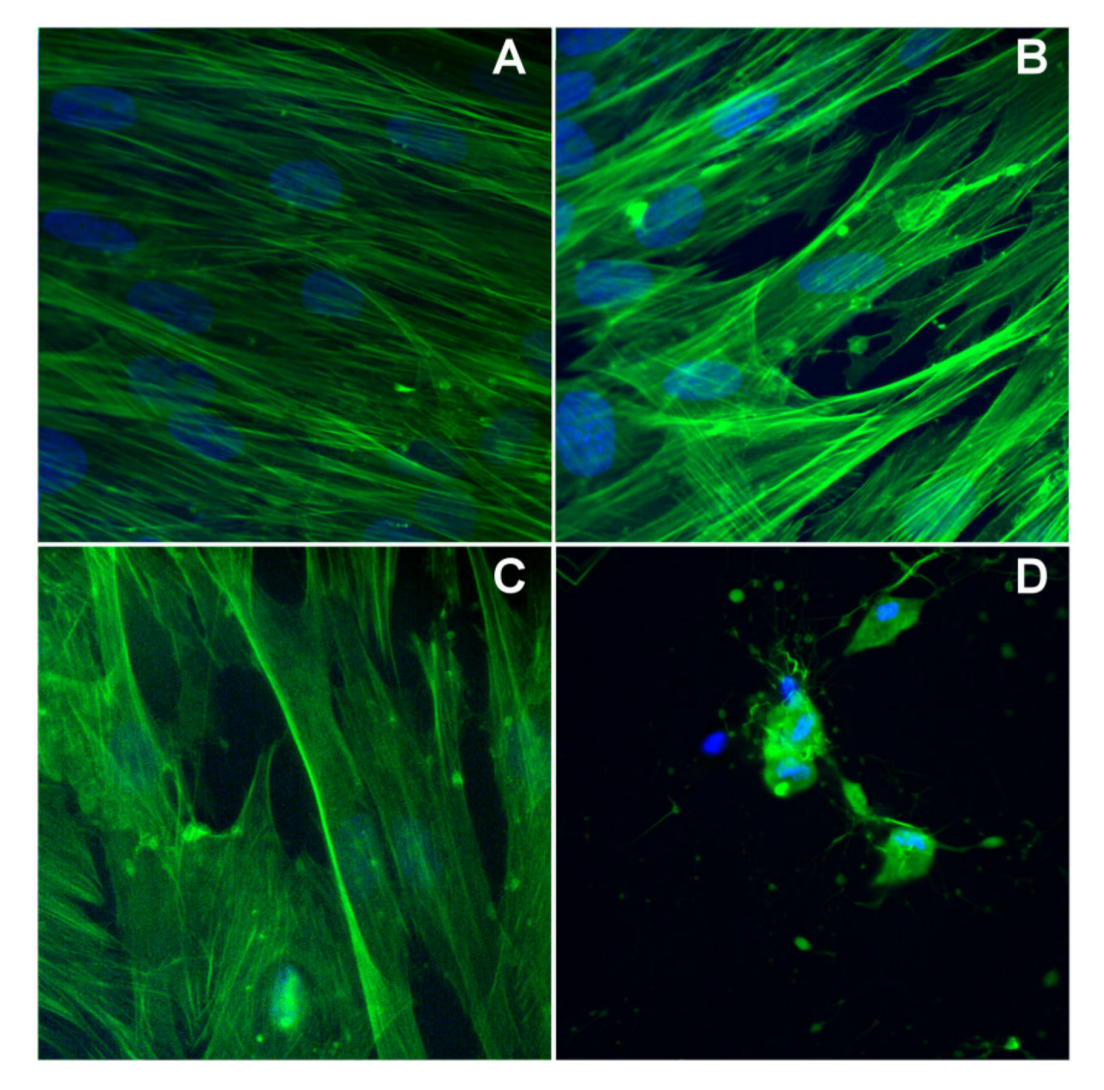
The cytoskeleton alteration for BJ cells: (**A**) control cells, (**B**) cells treated with pmtp, (**C**) cells treated with (**1**), (**D**) cells treated with (**2**).

In the case of BJ cells, the exposure for 24 h at a concentration of 6 μg/mL does not alter its morphology, as compared with the control ones, in the case of pmtp and (**1**), but instead complex (**2**) affects the cell morphology (Figure 9). Specifically, both cytoskeleton and nucleus are affected by altering the specific structure observed in control cells.

## 3. Materials and Methods

### 3.1. Reagents

The chemicals for synthesis of the complexes were purchased from Sigma-Aldrich (Darmstadt, Germany) (copper(II) perchlorate hexahydrate (≥99.99% trace metals basis), 2,2′-bipyridine (bpy, 99%), 1,10-phenanthroline (phen, 99%), 1-phenyl-1,3-butanedione (99%) and 3-amino-4H-1,2,4-triazole (96%)) and Merck (Darmstadt, Germany) (dibenzo-18-crown-6-ether, potassium superoxide) as reagent grade and were used as received, without further purification. The 5-phenyl-7-methyl-1,2,4-triazolo[1,5-*a*]pyrimidine (pmtp) was synthesized by a method reported in the literature [30].

### 3.2. Physical Measurements

A EuroEA elemental analyzer (Perkin Elmer, Waltham, MA, USA) was used for chemical analyses (for C, N and H). Fourier Transform Infrared spectroscopy (FTIR) spectra were recorded in KBr pellets with a Tensor 37 spectrometer (Bruker, Billerica, MA, USA) in the 400–4000 cm^−1^ range. UV-Vis spectroscopy was performed in solid state on a V 670 spectrophotometer (Jasco, Easton, MD, USA) with Spectralon as standard in 200–1500 nm range. The X-band Electron Paramagnetic Resonance (EPR) spectroscopy measurements were carried out with a continuous wave X-Band EMX plus EPR spectrometer (Bruker AXS GmbH, Karlsruhe, Germany) equipped with a Bruker X-SHQ 4119HS-W1 X-Band resonator. The measurement parameters for the X-Band measurements if not otherwise mentioned were set as follows: microwave frequency 9.879 GHz, microwave power 2 mW, modulation amplitude 0.1 mT, conversion time 26.67 ms, time constant 10.24 ms at one scan. For the Q-Band measurements, a continuous wave Q-band Bruker ELEXSYS E500Q EPR spectrometer together with an ER 5106 QT-W resonator was used. The measurement parameters for the Q-Band measurements if not otherwise mentioned were set as follows: microwave frequency 34.1507 GHz, microwave power 0.59 mW, modulation amplitude 0.1 mT, conversion time 40 ms, time constant 20.48 ms at one scan. All measurements were carried out at room temperature. Simultaneous Thermogravimetric/Differential Scanning Calorimetry—Mass spectroscopy (TG-DSC/MS) measurements were performed on a TGA/DSC1 Thermal Analyser (Mettler Toledo, Schwarzenbach, Switzerland), coupled to a Vacuum Thermostar Mass Spectrometer (Pfeiffer, Asslar, Germany) under the following conditions: temperature range from 25 to 800 °C, heating rate 5 °C min^−1^, dynamic air atmosphere with a flow rate of 100 mL min^−1^, sample mass around 5 mg, 150 μL platinum crucible; empty crucible served as a reference. Blank curve was subtracted. Evolved gasses were introduced into Mass Spectrometer via 75-cm-long heated capillary. X-ray diffraction measurements on single-crystal were performed on a IPDS II diffractometer (STOE, Darmstadt, Germany) operating with Mo-Kα (λ = 0.71073 Å) X-ray tube with graphite monochromator. The structure was solved by direct methods and refined by full-matrix least squares techniques based on *F^2^*. The non-H atoms were refined with anisotropic displacement parameters. Calculations were performed using the SHELX-2013 crystallographic software package. A summary of the crystallographic data and the structure refinement are presented in Table 1. Crystallographic data (excluding structure factors) have been deposited with the Cambridge Crystallographic Data Centre with CCDC reference numbers 2012013-2012014. These data can be obtained free of charge via http://www.ccdc.cam.ac.uk/conts/retrieving.html, or from the Cambridge Crystallographic Data Centre, 12 Union Road, Cambridge CB2 1EZ, UK; fax: (+44) 1223-336-033; or e-mail: deposit@ccdc.cam.ac.uk.

### 3.3. Synthesis and Characterization of the Complexes

[Cu(bpy)_2_(pmtp)](ClO_4_)_2_ (**1**): To a solution containing copper(II) perchlorate hexahydrate (0.186 g, 0.5 mmol) in 50 mL water, a solution containing bpy (0.156 g, 1 mmol) in 25 mL ethanol was added. This mixture was magnetically stirred at 50 °C for 2 h, until the color turned blue, and then a solution containing pmtp (0.186 g, 0.5 mmol) in 25 mL ethanol was added. The stirring of the reaction mixture continued for 4 h, while the blue color intensified. Crystals suitable for X-ray analysis were obtained after two weeks by slow evaporation of this solution. Yield: 78% (0.30 g). Analysis, found: C, 49.07; H, 3.25; N, 14.34%; calculated for CuC_32_H_26_N_8_O_8_Cl_2_ (M_w_: 785.05 g mol^−1^): C, 48.96; H, 3.34; N, 14.27%, IR (KBr pellet, cm^−1^): ν(CH), 3100 w, 3083 w; ν(C=N)+υ(C=C), 1634 m; ν(C=N)_trp_, 1603 m; υ(C=N)_pym_, 1585 m; ν_3_(ClO_4_), 1091 vs; ν_4_(ClO_4_), 624 m; ν(Cu-N), 442 w, 417 w, UV-Vis (solid, nm): π→π*, 290; CTLM, 345; d_xz_, d_yz_→ d_z2_, 615; d_xy_→ d_z2_, 675.

[Cu(phen)_2_(pmtp)](ClO_4_)_2_ (**2**): To a solution containing copper(II) perchlorate hexahydrate (0.186 g, 0.5 mmol) in 50 mL water, a solution containing phen (0.180 g, 1 mmol) in 25 mL ethanol was added. This mixture was magnetically stirred at 50 °C for 2 h, until the color turned green and then a solution containing pmtp (0.186 g, 0.5 mmol) in 25 mL ethanol was added. The stirring of the reaction mixture continued for 4 h, until a blue color was formed. Crystals suitable for X-ray analysis were obtained after two weeks by slow evaporation of this solution. Yield: 75% (0.30 g). Analysis, found: C, 52.03; H, 3.08; N, 13.48%; calculated for CuC_36_H_26_N_8_O_8_Cl_2_ (M_w_: 833.10 g mol^−1^): C, 51.90; H, 3.15; N, 13.45%, IR (KBr pellet, cm^−1^): ν(CH), 3095 w, 3063 w; ν(C=N) + ν(C=C), 1626 m; ν(C=N)_trp_, 1605 m; ν(C=N)_pym_, 1581 m; ν_3_(ClO_4_), 1092 vs; ν_4_(ClO_4_), 623 m; ν(Cu-N), 442 w, 417w, UV-Vis (solid, nm): π→π*, 265; CTLM, 360; d_xz_, d_yz_→d_z2_, 610; d_xy_→ d_z2_, 680.

### 3.4. Biological Characterization of Compounds

#### 3.4.1. Superoxide Scavenging Ability

The superoxide scavenging ability of the complexes was tested using the KO_2_ compound as a superoxide source combined with EPR spectroscopy. To carry out the experiments, 10 μM in DMSO solution of the complexes was mixed with different concentrations of KO_2_ in DMSO solution, and the EPR signal intensity changes were monitored. The KO_2_ was dissolved by complexation with dibenzo-18-crown-6-ether.

#### 3.4.2. Yeast Cell Experiments

The *Saccharomyces cerevisiae* laboratory strain BY4741 (*MAT***a**; *his3Δ1*; *leu2Δ0*; *met15Δ0*; *ura3Δ0*), considered wild type (WT), and the isogenic strains *sod1Δ* and *sod2Δ* lacking the genes *SOD1* (encoding Cu/Zn-superoxide dismutase) and *SOD2* (encoding Mn-superoxide dismutase) were purchased from EUROSCARF (Frankfurt, Germany). Cells were maintained and manipulated and grown in YPD medium (1% *w*/*v* yeast extract, 2% *w*/*v* peptone, 2% *w*/*v* glucose; 2% *w*/*v* agar was added for solid media) [44]. Complexes were added to autoclaved media from DMSO sterile stocks (10 mM). To determine the effect of compounds on yeast growth, a fresh overnight pre-culture was 10^3^-fold diluted in YPD and incubated (200 rpm, 30 °C) until the culture reached a density of 5 × 10^5^ cells/mL. At this point, the compounds were added to the desired concentrations, and cell proliferation was monitored at 660 nm [45] in a 96-well multi-scanner autoreader plate reader equipped with a thermostat and shaker (Varioskan Thermo Fischer Scientific, Vantaa, Finland). The uptake of compounds by yeast cells was determined indirectly by measuring the increase of the total cellular copper content [46] normalized to the total cellular protein determined by Bradford assay [47]. All determinations were done on three biological replicates. The IC_50_ was calculated by nonlinear regression analysis using the Graphpad Prism software.

#### 3.4.3. Spectroscopic Determination of the Compounds’ Interaction with DNA

To determine whether compounds have the capacity to interact with DNA, 48,500 base-pairs of genomic DNA from bacteriophage lambda (λ-DNA, Promega. Madison USA) was used. A stock solution of 10 µM λ-DNA (base-pair concentration) was prepared in 10 mM Tris/HCl buffer containing 1 mM NaCl, pH 8.

##### Effect of DNA on Spectral Characteristics of Compounds

The UV-Vis absorption titration experiments were done maintaining constant the complex concentration (3 µM) and increasing the concentration of λ-DNA, in Tris-HCl/NaCl buffer solution, at room temperature, until a saturation state was achieved [35,36]. After DNA addition, the solutions were allowed to equilibrate for 5 min at room temperature. The UV-Vis absorption spectra were recorded with a Jasco V-630 spectrophotometer, using a 10 mm quartz cell. Stock solutions of compounds were prepared in DMSO (1 mM) and diluted to 3 µM in 10 mM Tris/HCl containing 1 mM NaCl, pH 8.

##### Effect of Compounds on the Fluorescence of λ-DNA/Ethidium Bromide Adduct

The binding of compounds to λ-DNA was assayed by monitoring the quenching of fluorescence emitted by λ-DNA/ethidium bromide (EB) adduct (λ_excit_ = 510, maximum emission at λ_em_ = 601). The λ-DNA solution was added to the EB solution, prepared in the same buffer, then increasing concentrations of compounds were added to EB-DNA [2,48]. The fluorescence spectra were recorded in the range of 550–750 nm using a Thermo Fischer Scientific Varioskan Flash spectral scanning multimode reader (Vantaa, Finland). The spectra were recorded in suitable plates using 5 nm excitation and emission slits for all measurements.

#### 3.4.4. Nuclease Activity Assay

The nuclease activity of the compounds was determined using plasmid pRS325II DNA, which was a gift from Steven Haase (Addgene plasmid #35467, 6835 base pairs) [49]. The plasmid was amplified in *Escherichia coli* by transforming One Shot^®^ TOP10 chemically competent *E*. *coli* from Invitrogen, Thermo Fisher Scientific (Vilnius, Lithuania). Plasmid was isolated from positive colonies using a PureLink™ HiPure Plasmid Miniprep Kit from Invitrogen, Thermo Fisher Scientific (Vilnius, Lithuania). Plasmid (200 ng/sample) was exposed to 3 µM compounds in the presence of 1 mM hydrogen peroxide (to exploit the Cu(II/III) redox couple) or 1 mM ascorbic acid (to exploit the Cu(I/II) redox couple) [50]. The DNA cleavage experiments for the complexes were done in a 9/1 (*v*/*v*) ratio of 50 mM Tris-HCl buffer, pH 8, and DMSO. The samples were incubated for 1 h at 37 °C before a bromophenol blue/xylene cyanol-based loading dye (Roth, Germany) was added to the samples. Partial cleavage of the plasmid to linear form was done with the restriction enzyme *Eco*RI (Promega, USA) by incubation for 5 min at 37 °C. The samples were loaded on a 1% (*w*/*v*) agarose gel containing 1 μg/mL EtBr in 1 × TAE (Tris–acetic acid–EDTA) buffer. Electrophoresis was performed at 100 V for 45 min in 1 × TAE buffer. The images of the fluorescent ethidium bromide-stained gels were captured using a gel documentation system (Doc-Print II, Vilber Lourmat, France). The cleavage experiments were done six times, with similar results. One representative gel is shown.

#### 3.4.5. Screening of the Antibacterial Properties

The antibacterial assays were performed on Gram-negative (*Escherichia coli* ATCC 25922, *E. coli* 5, *Pseudomonas aeruginosa* ATCC 27853, *P. aeruginosa* ATCC 9027) and Gram-positive (*Staphylococcus aureus* ATCC 25923, *S. aureus* ATCC 6538, MRSA (Meticillin-resistant *S. aureus*) 388, ATCC, American Type Culture Collection) bacterial strains. Microbial suspensions (1.5 × 10^8^ CFU/mL; colony forming units (CFU)) corresponding to 0.5 McFarland density obtained from 15 to 18-h bacterial cultures developed on solid media were used. The antimicrobial activity was tested on Mueller–Hinton agar medium. The compounds were solubilized in DMSO and the starting stock solution had a concentration of 1.000 μg/mL.

The quantitative assay of the antimicrobial activity was performed by the liquid medium microdilution method, in 96-well multi-well plates, to establish the minimal inhibitory concentration (MIC). For this purpose, serial two-fold dilutions of the compounds ranging between 1.000 and 1.95 μg/mL were performed in a 200 μL volume of broth and each well was seeded with 50 μL microbial inoculums. Sterility control (wells containing only culture medium) and culture controls (wells containing culture medium seeded with the microbial inoculum) were used. The influence of the DMSO solvent was also quantified in a series of wells containing DMSO, diluted accordingly with the dilution scheme used for the complexes. The plates were incubated for 24 h at 37 °C, and MIC values were considered as the lowest concentration of the tested compound that inhibited the visible growth of the microbial overnight cultures.

The assessment of the complexes’ influence on the microbial ability to colonize an inert substratum with the establishment of the minimal biofilm eradication concentration (MBEC) was performed by the micro-titer method, following previously described protocols [51]. The absorbance at 490 nm was measured with an Apollo LB 911ELISA (Berthold Technologies GmbH & Co. KG, Waltham, MA, USA) reader. All experiments were performed in triplicates.

#### 3.4.6. In Vitro Cytotoxicity Assay

##### Cell Culture Conditions

Human fibroblast cells (BJ-ATCC CRL-2522, Manassas, VA, USA) were grown in minimal essential medium (MEM) supplemented with 2 mM L-Glutamine, 10% fetal calf serum (FCS), 100 units/mL of penicillin and 100 µg/mL of streptomycin at 37 °C in a humidified incubator under an atmosphere containing 5% CO_2_. Mouse melanoma cells (B16-ATCC CRL-6475, Manassas, VA, USA) were grown in DMEM (Dulbecco’s Modified Eagle Medium) supplemented with similar reagents as MEM. All cell cultivation media and reagents were purchased from Biochrom AG (Berlin, Germany) and Sigma-Aldrich (Darmstadt, Germany).

##### Cell Viability Assay

Cell viability was evaluated using 3-(4,5-dimethylthiazol-2-yl)-2,5-diphenyltetrazolium bromide (MTT) assay, as described previously [52]. First, the cells were seeded in 96-well plates (20,000 cells/well) and cultured for 24 h in medium. After overnight incubation, the medium was changed and the investigated sample in concentration varying from 0.03 to 0.25 mg/mL was added for 24 h. The negative control was represented by cells cultivated in medium without the investigated compounds. Following incubation, the medium was changed and the MTT solution was added to each well to a final concentration of 1 mg/mL and incubated for additional 4 h, at 37 °C. Finally, the medium was collected and DMSO was used to dissolve the insoluble formazan product. The absorbance of the samples was recorded at 570 nm using a plate reader Mithras 940 (Berthold). The data were corrected for the background and the percentage of viable cells was obtained using the equation:

Cell viability = [(A_570_ of treated cells)/(A_570_ of untreated cells)] × 100%

The half-maximal inhibitory concentration (IC_50_) was determined by fitting the data with a logistical sigmoidal equation using the software Origin 8.1 from Microcal Inc. (Los Angeles, CA, USA).

##### Measurement of the Hemolytic Activity

The hemolytic activity of the compounds was determined using an adapted protocol of ASTM F 756–00 standard [38,53] based on hemoglobin release from human red blood cells (hRBCs) resulting after cell lyses. Briefly, the blood samples were collected from healthy volunteers, diluted to a concentration of hemoglobin ~10 mg/mL with phosphate buffer saline (PBS) and then incubated with various concentrations of compounds for 4 h at 37 °C. After 4 h, the cells were centrifuged, the supernatant was collected, transferred into 96-well tissue culture plates and mixed with Drabkin reagent, which converts hemoglobin to cyanmethemoglobin (absorption peak at 540 nm). After 15 min, the absorbance of the samples was read at 570 nm using a plate reader. The obtained data was background corrected and used to calculate the hemolytic index as percentage of the hemoglobin released after the treatment with the tested compounds, relative to control samples. The hRBCs in PBS and distilled water were used as negative and positive controls.

##### Phalloidin Staining and Cell Imaging

Cytoskeleton actin filaments of BJ and B16 cells were stained with phalloidin-FITC (Sigma-Aldrich, USA) according to the manufacturer’s protocol. Briefly, cells were washed with PBS (5 min, 3 times), fixed for 5 min with 3% paraformaldehyde, washed three times with PBS, permeabilized with 0.1% Triton X-100 in PBS for 15 min, washed three times with PBS, stained with 20 μg mL^−1^ phalloidin-FITC at room temperature for 1 h and washed again three times with PBS. Cell nucleus was stained with 8 μM of Hoechst 33342 solution for 10 min, washed three times with PBS and finally, mounted and sealed on glass slides with FluorSave™ Reagent (Merck Millipore, Germany). The fluorescence images were acquired using an Andor DSD2 Confocal Unit (Andor, Ireland), mounted on an epifluorescence microscope, Olympus BX-51 (Olympus, Germany), equipped with a 40x and 100x objective and an appropriate DAPI/Hoechst filter cube (excitation filter 390/40 m, dichroic mirror 405 nm and emission filter 452/45 nm) and GFP/FITC filter cube (excitation filter 466/40 nm, dichroic mirror 488 nm and emission filter 525/54 nm).

## 4. Conclusions

Novel copper(II) complexes with a N-N-chelating heterocycle (2,2′-bipyridine or 1,10-phenanthroline) and 5-phenyl-7-methyl-1,2,4-triazolo[1,5-*a*]pyrimidine were synthesized, and structurally and biologically characterized. Although the two compounds have a similar formula [Cu(N-N)_2_pmtp](ClO_4_)_2_, the packing pattern in solid phase is different as a result of π-π stacking interactions realized between both pmtp and 1,10-phenanthroline ligands.

Complexes are very stable in DMSO solution over a long period and exhibit superoxide scavenging abilities, as indicated by EPR spectroscopy.

The hypochromic effect observed in absorption maxima located in UV region upon spectrometric titrations of λ-DNA shows the complexes’ intercalative abilities to be mediated by the aromatic rings of ligands.

Complex (**2**) exhibits an intrinsic nuclease activity and both complexes display a strong nuclease activity manifested by plasmid DNA relaxation into its nicked-circular form, a process promoted by the reactive species generated in the presence of ascorbate or hydrogen peroxide.

Both complexes exhibited improved antimicrobial activity, as compared to the ligand. Complex (**2**) was by far more active than (**1**) against the Gram-positive and Gram-negative strains, susceptible or resistant, growing in suspension or in biofilms. Both MIC and MBEC values decreased in the case of complex (**2**) by 8 to 40 times compared to (**1**).

Although the studies performed on *Saccharomyces cerevisiae*, BJ and human red blood cells do not indicate any significant toxicity in the concentration ranges proved for manifestation of biological activity, the results obtained by in vitro studies on melanoma tumor cells (B16) support the potential of the new complexes as anticancer drugs. Complex (**1**) was more susceptible than (**2**), as revealed by both therapeutic index value and morphological (cytoskeleton and nucleus) modifications.

Our results suggest that both antimicrobial and antitumor activity of the copper(II) complexes involves intercalative interaction with DNA as well as DNA damage through the metallonuclease activity of complexes.

## Figures and Tables

**Table 1:** Crystal data and structure refinement for compounds (**1**) and (**2**).

Compound	(1)	(2)
Empirical formula	C_32_H_26_Cl_2_CuN_8_O_8_	C_37_H_27_Cl_2_CuN_7_O_8_
Formula weight	785.05	832.09
Temperature/K	293	293
Crystal system	monoclinic	triclinic
Space group	*P*a	*P*-1
a/Å	19.4489(2)	9.0469(2)
b/Å	8.1324(3)	13.4858(3)
c/Å	21.7298(4)	14.3149(4)
α/°	90	93.628(3)
β/°	102.445(3)	95.081(2)
γ/°	90	95.071(4)
Volume/Å^3^	3356.16(15)	1728.36(7)
Z	4	2
ρ_calc_g/cm^3^	1.554	1.599
μ/mm^−1^	0.874	0.853
F(000)	1604.0	850.0
Radiation	MoKα (λ = 0.71073)	MoKα (λ = 0.71073)
Reflections collected	15462	20217
Independent reflections	9420 [R_int_ = 0.0933, R_sigma_ = 0.0767]	6042 [R_int_ = 0.0719, R_sigma_ = 0.0489]
Data/restraints/parameters	9420/2/921	6042/7/498
Goodness-of-fit on F^2^	1.065	1.126
Final R indexes [I ≥ 2σ (I)]	R_1_ = 0.0910, wR_2_ = 0.2222	R_1_ = 0.0639, wR_2_ = 0.1782
Final R indexes [all data]	R_1_ = 0.1436, wR_2_ = 0.2679	R_1_ = 0.0721, wR_2_ = 0.1885
Largest diff. peak/hole/e Å^−3^	2.66/−0.61	1.794/−1.076
Flack parameter	−0.04(4)	

**Table 2:** Selected bond distances (Å) and bond angles (°) for (**1**) and (**2**).

(1)							(2)						
Cu1	N2	1.971(15)	N2	Cu1	N4	176.8(7)	Cu1	N2	1.999(4)	N2	Cu1	N4	173.26(14)
Cu1	N4	1.968(16)	N2	Cu1	N1	80.6(6)	Cu1	N4	2.001(3)	N2	Cu1	N1	95.30(15)
Cu1	N1	2.112(16)	N2	Cu1	N5	90.8(6)	Cu1	N1	2.020(3)	N2	Cu1	N3	81.11(13)
Cu1	N5	2.013(14)	N2	Cu1	N3	95.8(7)	Cu1	N3	2.045(3)	N2	Cu1	N5	97.20(12)
Cu1	N3	2.072(18)	N4	Cu1	N1	98.8(6)	Cu1	N5	2.203(3)	N4	Cu1	N1	91.29(14)
Cu2	N12	2.071(17)	N4	Cu1	N5	92.1(6)				N4	Cu1	N3	93.70(12)
Cu2	N9	1.995(15)	N4	Cu1	N3	81.4(7)				N4	Cu1	N5	79.49(12)
Cu2	N13	1.958(15)	N5	Cu1	N1	121.9(6)				N1	Cu1	N3	150.86(12)
Cu2	N10	1.987(15)	N5	Cu1	N3	130.0(6)				N1	Cu1	N5	108.61(13)
Cu2	N11	2.056(16)	N3	Cu1	N1	108.0(7)							
			N9	Cu2	N12	122.2(7)							
			N9	Cu2	N11	129.6(6)							
			N13	Cu2	N12	81.2(7)							
			N13	Cu2	N9	90.6(6)							
			N13	Cu2	N10	176.9(7)							
			N13	Cu2	N11	97.7(7)							
			N10	Cu2	N12	98.8(7)							
			N10	Cu2	N9	92.0(6)							

**Table 3:** The MIC values (mM) for ligand and complexes.

Bacterial Strain	pmtp	bpy	*CuBP	(1)	phen	**CuPP	(2)
*E. coli* 25922	3.00	4.00	0.54	0.20	3.50	0.03	0.02
*E. coli* 5 ESBL	3.00	4.00	0.54	0.40	3.50	0.03	0.05
*P. aeruginosa* 27853	3.00	8.00	1.09	0.40	7.00	0.50	0.09
*P. aeruginosa* 9027	3.00	4.00	1.09	0.40	3.50	0.50	0.09
*S. aureus* 25923	6.00	8.00	0.27	0.40	7.00	0.03	0.01
*S. aureus* 6538	3.00	8.00	0.27	0.40	7.00	0.03	0.09
MRSA	6.00	8.00	0.27	0.40	7.00	0.12	0.05

*CuBP: {[Cu(bpy)_2_(μ_2_OClO_3_)]·ClO_4_}n; **CuPP: [Cu(phen)_2_(OH_2_)](ClO_4_)_2_.

**Table 4:** The compounds influence on biofilm formation (MBEC value, mM).

Bacterial Strain	pmtp	bpy	CuBP	(1)	phen	CuPP	(2)
*E. coli* 25922	6.00	8.00	0.54	0.40	7.00	0.10	0.02
*E. coli* 5 ESBL	3.00	8.00	0.54	0.40	3.50	0.30	0.05
*P. aeruginosa* 27853	3.00	8.00	1.09	0.40	7.00	0.50	0.09
*P. aeruginosa* 9027	1.50	4.00	1.09	0.20	3.50	0.50	0.01
*S. aureus* 25923	6.00	4.00	0.14	0.80	7.00	0.03	0.01
*S. aureus* 6538	3.00	8.00	0.14	0.10	7.00	0.03	0.02
MRSA	6.00	8.00	0.27	0.40	7.00	0.10	0.05

**Table 5:** The half-maximal inhibitory concentration (IC_50_) and hemolysis data for compounds.

Compound	IC_50_ (µg/mL; mM)				Hemolysis (%)	TI	
	B16 Cells		BJ Cells				
	24 h	48 h	24 h	48 h		24 h	48 h
pmtp	ND	ND	ND	ND	0.61 ± 0.05	-	-
(**1**)	ND	15.46; 0.02	ND	ND	0.59 ± 0.18	-	3.23
(**2**)	6.25; 0.0075	3.72; 0.004	5.98; 0.007	3.30; 0.004	0.77 ± 0.14	0.95	0.88

ND: not determined; TI: therapeutic index.

## Supplementary Materials

The following are available online. Figure S1: UV-Vis spectra of complex (**1**) (dark blue) and complex (**2**) (light blue). Figure S2. EPR spectra of complex (**1**) and (**2**) in DMSO. Figure S3. EPR spectra (registered and simulated) of complex (**1**) and (**2**) in DMSO. Figure S4. TG and DSC curves for complexes. Figure S5. TG curve together with evolved gases for complex (**1**). Figure S6. EPR spectra of complex (**1**) and (**2**) in interaction with superoxide. Figure S7. Effect of compounds (**1**) and (**2**) on *Saccharomyces cerevisiae* (effect on growth yeast cell proliferation determined after 16 h of exposure to various concentrations of complexes expressed relatively to cell growth in the absence of compounds (**A**), effect of compounds on growth of superoxide dismutase-deficient mutants *sod1Δ* and *sod2Δ* (**B**) and compounds’ uptake expressed as copper accumulation (**C**)). Table S1. Continuous shape measure for the coordination polyhedron around the Cu(II) atom. Table S2. Thermal data for complexes in air atmosphere.
