# Supplementary material for: Copper(II) Complexes with Mixed Heterocycle Ligands as Promising Antibacterial and Antitumor Species
Source: Molecules. 2020 Aug 19;25(17):3777. doi: 10.3390/molecules25173777 (PMC7504215; doi:10.3390/molecules25173777)
Supplement: Supplementary file 1 [file molecules-25-03777-s001.pdf]

Copper(II) Complexes with Mixed Heterocycle Ligands as Promising Antibacterial and Antitumor Species

Arpad Mihai Rostas, Mihaela Badea, Lavinia L. Ruţă, Ileana C. Farcaşanu, Cătălin Maxim, Mariana Carmen Chifiriuc, Marcela Popa, Mirela Luca, Nataša Čelan Korošin, Romana Cerc Korošec, Mihaela Bacalum, Mina Răileanu and Rodica Olar*

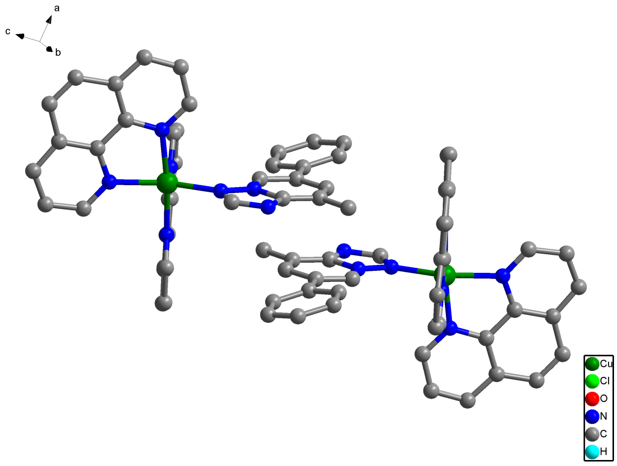

Figure S1. Supramolecular dimers of [Cu(phen)$_2$(pmtp)](ClO$_4$)$_2$ (**2**) generated through intermolecular π-π stacking interactions.

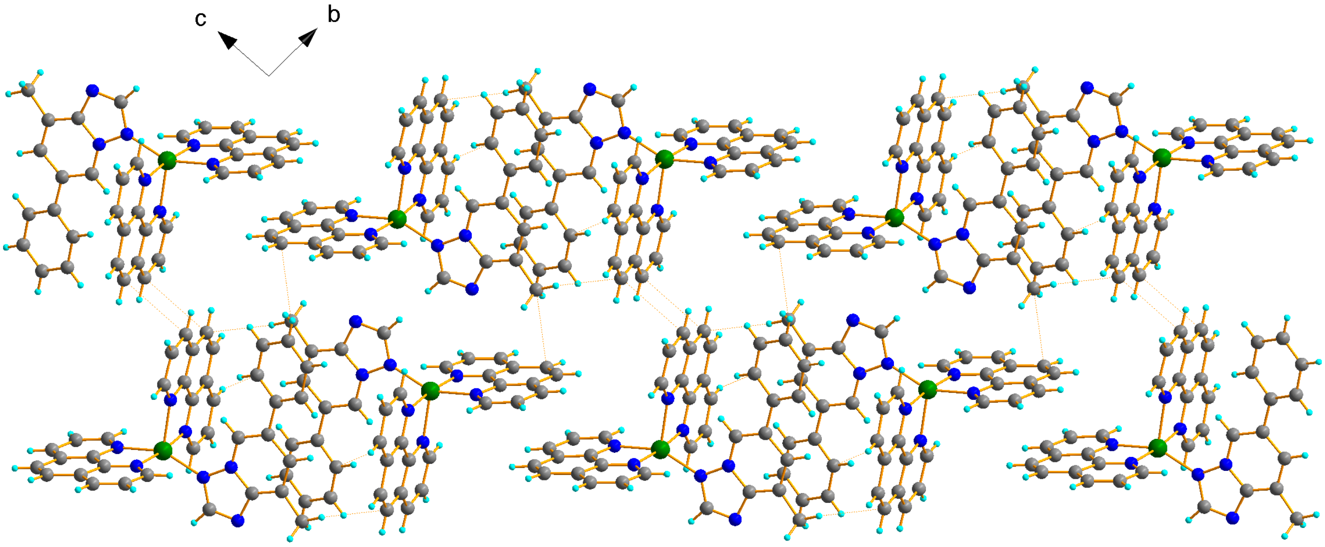

Figure S2. Packing diagram of [Cu(phen)$_2$(pmtp)](ClO$_4$)$_2$ (**2**) showing the supramolecular chains along a axes.

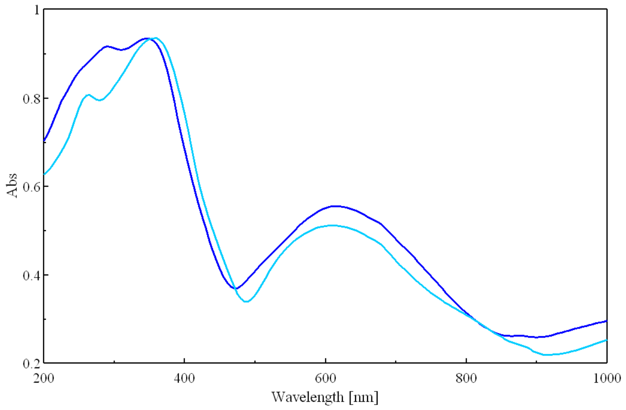

Figure S3. UV-Vis spectra of complex (**1**) (dark blue) and complex (**2**) (light blue).

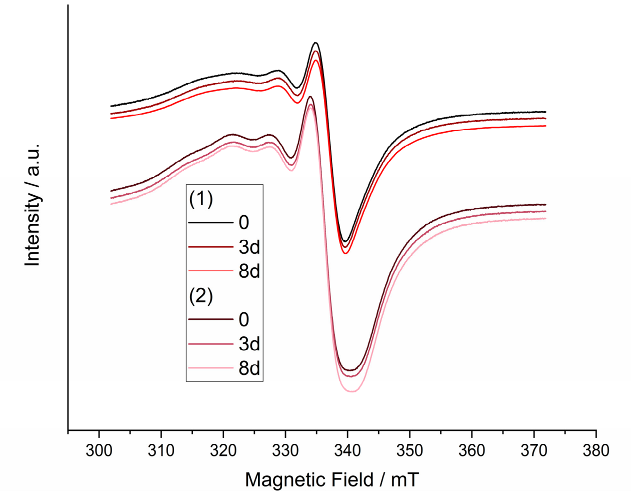

Figure S4. EPR spectra of complex (**1**) and (**2**) in DMSO.

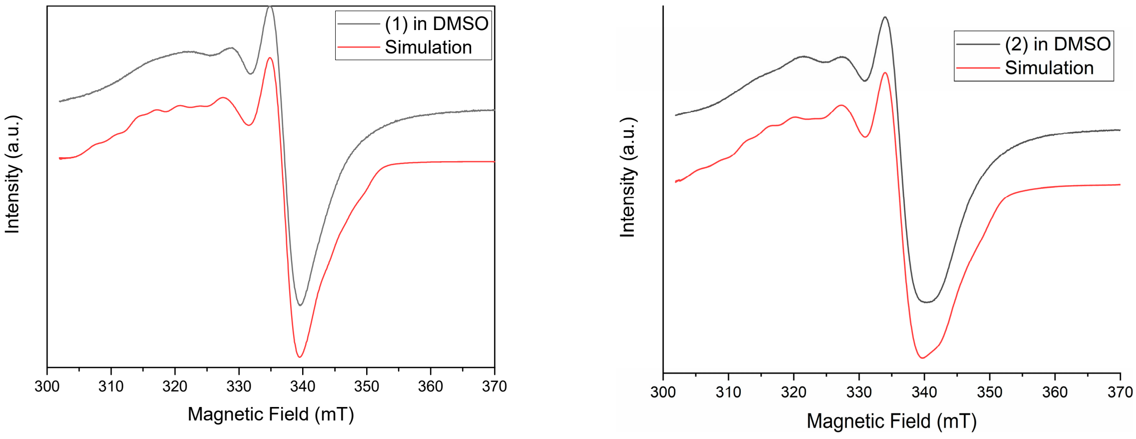

Figure S5. EPR spectra (registered and simulated) of complex (**1**) and (**2**) in DMSO.

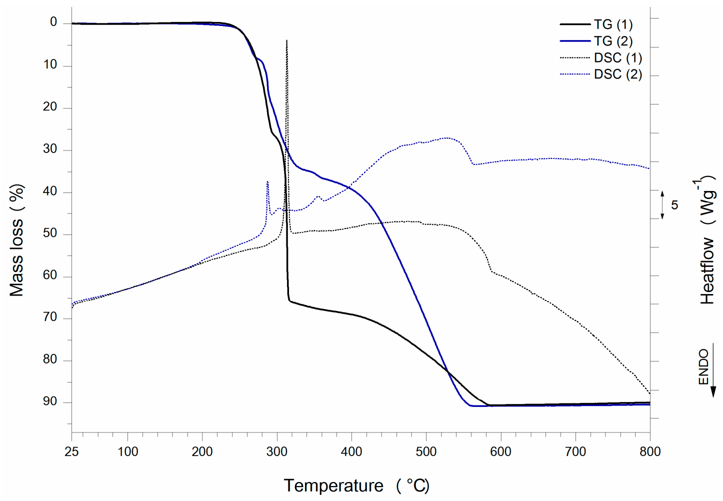

Figure S6. TG and DSC curves for complexes.

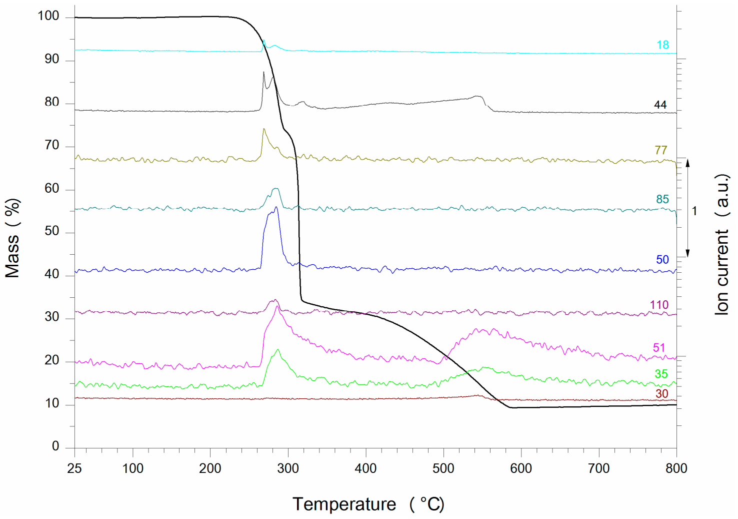

Figure S7. TG curve together with evolved gases for complex (**1**).

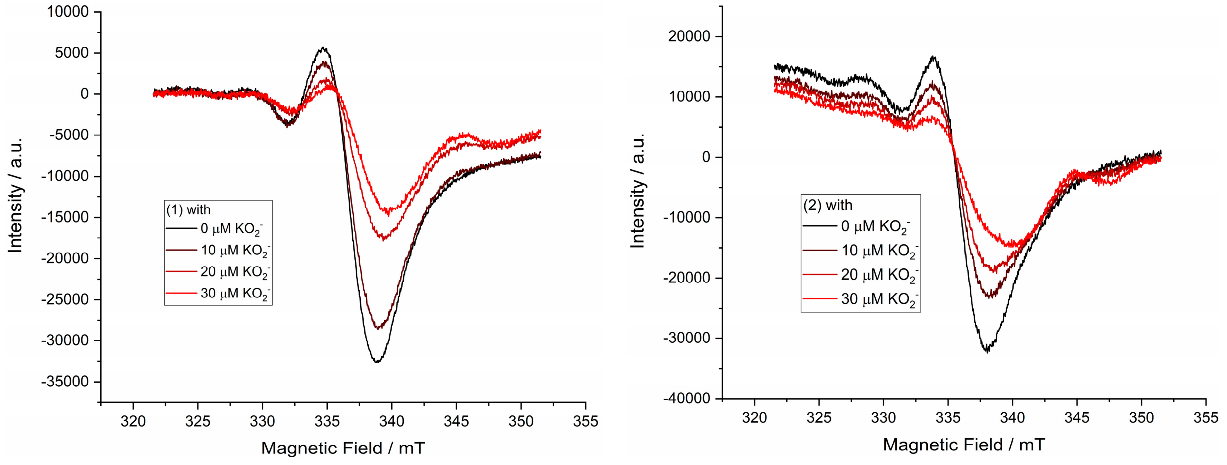

Figure S8. EPR spectra of complex (**1**) and (**2**) modification in interaction with superoxide.

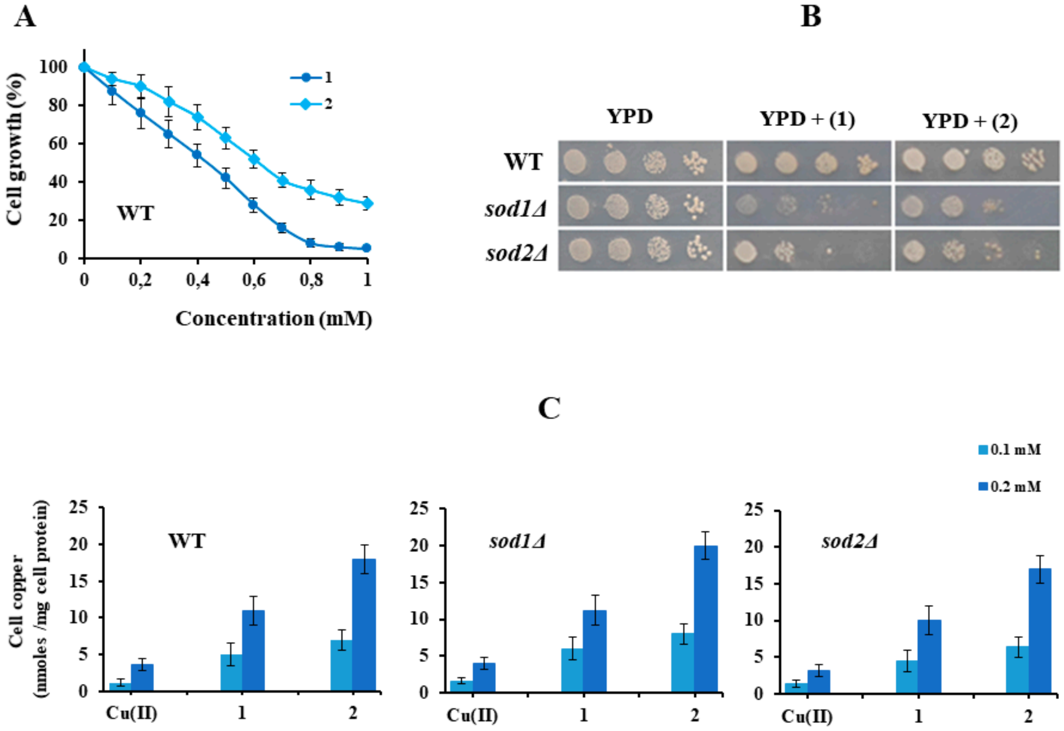

Figure S9. Effect of compounds (**1**) and (**2**) on *Saccharomyces cerevisiae* (effect on growth yeast cell proliferation determined after 16 hours of exposure to various concentrations of complexes expressed relatively to cell growth in the absence of compounds (**A**), effect of compounds on growth of superoxide dismutase-deficient mutants *sod1Δ* and *sod2Δ* (**B**) and compound's uptake expressed as copper accumulation (**C**)).

Table S1. Continuous shape measure for the coordination polyhedron around the Cu(II) atom.

Geometry	Cu1 (compound 1)	Cu2 (compound 1)	Cu1 (compound 2)
PP-5	29.381	28.943	29.021
vOC-5	5.897	6.019	2.779
TBPY-5	0.891	0.957	2.277
SPY-5	4.307	4.487	2.381
JTBPY-5	4.513	4.331	5.488

Table S2. Thermal data for complexes in air atmosphere.

**Compound**	**Step**	**Thermal effect**	**Temperature range / °C**	**Δm$_{exp}$ /%**	**Identified product by EGA with corresponding *m*/*z* values**
(**1**)	1.	Exothermic	198-294	27.00	moieties from pmtp (15 (CH_3), 60, 61 ($C_2H_9N_2$), 77, 78 (C_6H_5), 85 ($C_2H_5N_4$), 109, 110 ($C_5H_8N_3$)), moieties from dipy (80 (C_5H_6N), 112, 113 ($C_6H_{13}N_2$)), moieties from perchlorate (Cl (35), CHCl (49), ClO (50, 51), Cl_2 (70)), H_2O (18), CO_2 (44)
	2.	Exothermic	294-356	41.15	moieties from perchlorate (Cl (35), CHCl (49), ClO (50, 51), ClO_2 (69), Cl_2 (70), HCl_2 (72)), 44(CO_2)
	3.	Exothermic	356-660	22.68	moieties from perchlorate (Cl (35), ClO (51), ClO_2 (69), Cl_2 (70), HCl_2 (72)), 30 (NO), 44(CO_2)
(**2**)	1.	Exothermic	195-266	8.18	moieties from pmtp (15 (CH_3), 77, 78 (C_6H_5), 85 ($C_2H_5N_4$), 110 ($C_5H_8N_3$)), moieties from dipy (112, 113 ($C_6H_{13}N_2$)), moieties from perchlorate (Cl (35), CHCl (49), ClO (50, 51), Cl_2 (70))
	2.	Exothermic	266-287	12.17	moieties from pmtp (15 (CH_3), 77, 78 (C_6H_5), 85 ($C_2H_5N_4$), 110 ($C_5H_8N_3$)), moieties from dipy (112, 113 ($C_6H_{13}N_2$)), moieties from perchlorate (Cl (35), CHCl (49), ClO (50, 51), Cl_2 (70)), H_2O (18), 44(CO_2)
	3.	Exothermic	287-358	16.43	ClO (50, 51), 30 (NO), 44(CO_2)
	4.	Exothermic	358-570	53.96	ClO (50, 51), 30 (NO), 44(CO_2)
